# Examining Sleep Modulation by *Drosophila* Ellipsoid Body Neurons

**DOI:** 10.1523/ENEURO.0281-23.2023

**Published:** 2023-09-25

**Authors:** Prabhjit Singh, Abigail Aleman, Jaison Jiro Omoto, Bao-Chau Nguyen, Pratyush Kandimalla, Volker Hartenstein, Jeffrey M. Donlea

**Affiliations:** 1 Department of Neurobiology, David Geffen School of Medicine, University of California–Los Angeles, Los Angeles, California 90095; 2 Molecular, Cellular & Integrative Physiology Interdepartmental Program, University of California–Los Angeles, Los Angeles, California 90095; 3 Department of Molecular, Cell, & Developmental Biology, University of California–Los Angeles, Los Angeles, California 90095

**Keywords:** *Drosophila*, sleep

## Abstract

Recent work in *Drosophila* has uncovered several neighboring classes of sleep-regulatory neurons within the central complex. However, the logic of connectivity and network motifs remains limited by the incomplete examination of relevant cell types. Using a recent genetic–anatomic classification of ellipsoid body ring neurons, we conducted a thermogenetic screen in female flies to assess sleep/wake behavior and identified two wake-promoting drivers that label ER3d neurons and two sleep-promoting drivers that express in ER3m cells. We then used intersectional genetics to refine driver expression patterns. Activation of ER3d cells shortened sleep bouts, suggesting a key role in sleep maintenance. While sleep-promoting drivers from our mini-screen label overlapping ER3m neurons, intersectional strategies cannot rule out sleep regulatory roles for additional neurons in their expression patterns. Suppressing GABA synthesis in ER3m neurons prevents postinjury sleep, and GABAergic ER3d cells are required for thermogenetically induced wakefulness. Finally, we use an activity-dependent fluorescent reporter for putative synaptic contacts to embed these neurons within the known sleep-regulatory network. ER3m and ER3d neurons may receive connections from wake-active Helicon/ExR1 cells, and ER3m neurons likely inhibit ER3d neurons. Together, these data suggest a neural mechanism by which previously uncharacterized circuit elements stabilize sleep–wake states.

## Significance Statement

Neural circuits that control sleep must be stable to ensure therapeutic rest but readily adaptable to a variety of experiences. The *Drosophila* ellipsoid body (EB) regulates many cognitive processes, and recent studies show that certain EB neurons can intensify sleepiness, but the contributions of other EB neuron subclasses in sleep regulation remain unclear. Thus, we searched for sleep-regulatory neurons across the EB to better understand sleep control. Our studies indicate that ER3d neurons promote wakefulness by reducing the persistence of sleep episodes and suggest that ER3m neuron activity may increase sleep. Understanding the stability and flexibility of sleep, then, may emerge from investigating the connectivity and conductivity of sleep-regulatory neurons in the EB.

## Introduction

Sleep is a vital state to maintain physiology and neural processing (Everson et al., 1989; Everson, 1995; Shaw et al., 2002; Dongen et al., 2003; Vaccaro et al., 2020), but the organization of sleep control circuits is not fully understood in any species. Previous *Drosophila* studies have identified multiple neuronal populations in the central complex that regulate sleep. This circuit includes one subclass of ER neurons (a category of ring neurons), that innervates the anterior domain of the ellipsoid body (EB; Liu et al., 2016), a toroid structure along the midline of the fly brain involved in spatial orientation, navigation, motor control, and arousal (Bausenwein et al., 1994; Lebestky et al., 2009; Ofstad et al., 2011; Seelig and Jayaraman, 2015; Fisher et al., 2019; Kim et al., 2019; Kottler et al., 2019). The EB is composed of several cell populations, including innervation from anatomically distinct types of ring neurons characterized by their circular arbors (Hanesch et al., 1989; Renn et al., 1999; Young and Armstrong, 2010; Lin et al., 2013; Omoto et al., 2018). They can be subdivided into ER and ExR neurons based on their developmental origins (Hanesch et al., 1989), and into unique subclasses based on morphology (Omoto et al., 2018) and connectivity (Hulse et al., 2021). Each ER neuron typically contains dendrites lateral to the EB, generally in the bulb, then projects an axon in a concentric annulus within the EB (Hanesch et al., 1989; Omoto et al., 2018). The EB colocalizes with strong GABA immunostaining (Hanesch et al., 1989; Enell et al., 2007; Zhang et al., 2013; Xie et al., 2017; Shaw et al., 2018) and is labeled by *gad1*-Gal4 (Enell et al., 2007; Kahsai and Winther, 2011), suggesting that many ER neurons may be GABAergic.

Recent experiments identified an ER neuron subclass that becomes active with waking experience and can drive a persistent sleep increase on activation (Liu et al., 2016). These neurons were named “R2” when their role in sleep drive was described (Liu et al., 2016; Donlea et al., 2018), but have since been called “R5/ER5” (Omoto et al., 2018; Raccuglia et al., 2019; Blum et al., 2021; Hulse et al., 2021) to avoid conflation with other ER neurons that were designated R2 (Hanesch et al., 1989; Seelig and Jayaraman, 2013; Omoto et al., 2017; Fisher et al., 2019). Sleep-related inputs may reach ER neurons by at least three pathways, as follows: visual projection tuberculo-bulbar neurons, arousal-encoding Helicon/ExR1 neurons, and circadian circuitry via dopaminergic interneurons (Donlea et al., 2018; Guo et al., 2018; Lamaze et al., 2018; Omoto et al., 2018; Liang et al., 2019; Raccuglia et al., 2019; Hulse et al., 2021). Serotonergic modulation of ER neuron populations influences sleep architecture (Liu et al., 2019), but the roles of many ER neurons on sleep/wake regulation remain undescribed.

To test whether other ER neurons influence sleep, we conducted a thermogenetic activation screen using the warm-sensitive cation channel *TrpA1* (Hamada et al., 2008). We subsequently pursued two arousal-inducing lines that express in ER3d neurons, and two sleep-promoting drivers that label ER3m cells. Refined driver intersections support a wake-promoting role for ER3d neurons, whose activation impairs sleep maintenance. Further characterization of sleep-promoting drivers suggests a role for ER3m neurons, but also indicates that other neuron types labeled by these drivers may contribute to sleep regulation. Both ER3m and ER3d populations colocalize with GABA immunostaining; Gad1 knockdown in ER3m neurons prevents flies from increasing sleep following neural injury, and wake-promoting effects of ER3d-drivers rely on GABAergic neurons. To better characterize connectivity between ER3m, ER3d, and previously identified sleep-regulatory EB cell types, we imaged contacts labeled by a genetically encoded synaptic reporter (Macpherson et al., 2015). We find that ER3m and ER3d neurons receive putative synaptic inputs from arousal-promoting Helicon/ExR1 neurons but form few connections with ER5 cells. Together, these results suggest novel sleep regulatory effects for two ER neuron subclasses that project into the anterior half of the EB: activation of ER3m neurons increases sleep while ER3d stimulation promotes wakefulness.

## Materials and Methods

### Experimental model and subject details: fly stocks and maintenance

Fly stocks were reared on standard cornmeal media (per 1 L of H_2_O: 12 g agar, 29 g Red Star yeast, 71 g cornmeal, 92 g molasses, 16 ml of methyl paraben 10% in EtOH, 10 ml of propionic acid 50% in H_2_O) at 25°C with 60% relative humidity and entrained to a daily 12 h light/dark schedule. Canton-S flies were provided by Gero Miesenböck (University of Oxford, Oxford, UK), and UAS-*TrpA1* (Hamada et al., 2008) were from Paul Shaw (Washington University in St. Louis, St. Louis, MO).

ER neuron drivers were selected from previous studies (Omoto et al., 2017, 2018) and from visual inspection of publicly available confocal *z*-stacks shared by the FlyLight Project at the Howard Hughes Medical Institute Janelia Research Campus (https://flweb.janelia.org; Jenett et al., 2012). ER neuron identities in drivers were classified using the anatomic criteria defined in Omoto et al. (2018), which distinguishes ER neuron classes based on the following: (1) approximate quantity of soma; (2) regional positioning of microglomerular dendrites in the bulb or thin branches along the lateral face of the lateral accessory lobe; (3) centrifugal/inside-out or centripetal/outside-in route taken by the neurite into the EB; (4) radial projection of fibers in the EB (lateral/outer or medial/inner ring); (5) proximity to anterior or posterior faces of the EB; and, where appropriate, (6) arc of axonal projections in N-cadherin (N-cad) density domains of EB as revealed by dorsal mount preparations.

The following genetic driver lines were created for the Janelia Research Campus stock collection (Pfeiffer et al., 2010; Jenett et al., 2012; Dionne et al., 2018) and were ordered from the Bloomington Drosophila Stock Center (BDSC): *R12B01*-Gal4 (48487), *R12G08*-Gal4 (47855), *R28D01*-Gal4 (47342), *R28E01*-Gal4 (49457), *R31A12*-Gal4 (49661), *R34D03*-Gal4 (49784), *R37E10*-Gal4 (48132), *R47D08*-Gal4 (50305), *R58H05*-Gal4 (39198), *R59B10*-Gal4 (39209), *R78B06*-Gal4 (48343), *R80C07*-Gal4 (40074), *R84H09*-Gal4 (47803), *R92A09*-Gal4 (40598), *R24B11*-LexA (53547), *R28E01*-LexA (53523), *R48H04*-LexA (53609), *R54B05-*Gal4.DBD (69148), *R80C07-*p65.AD (70817), *R42D11*-p65.AD (75730), *R24B11*-p65.AD (70603), *R28E01-*p65.AD (70169), *R28E01*-Gal4.DBD (69109), *R47D08*-p65.AD (71067), *R92A09-*p65.AD (70849), and *R78A01*-Gal4.DBD (69876). The following are Gal4 lines generated for the Vienna Tiles library (Tirian and Dickson, 2017) and were shared by the Janelia Research Campus: *VT002226*-Gal4, *VT004309*-Gal4, *VT016270*-Gal4, *VT020036*-Gal4, *VT020036*-Gal4, *VT020613*-Gal4, *VT026873*-Gal4, *VT029750*-Gal4, *VT038873*-Gal4, *VT042805*-Gal4, *VT057232*-Gal4, *VT063740*-Gal4, *VT063949*-Gal4, *VT020613-*p65.AD (stock #75252, BDSC), *VT01968*-p65.AD (stock #71432, BDSC), *VT042805-*p65.AD (stock #74505, BDSC), and *VT042805-*Gal4.DBD (stock #75140, BDSC).

Gad1-KI-Gal80 were shared by Drs. Bowen Deng and Yi Rao (Peking University, Beijing, People’s Republic of China), Tsh*-*Gal80/cyo and TubP-FSF-Gal80;Tsh-LexA>LexAOP-Flp flies were a gift from Julie Simpson (University of California, Santa Barbara), UAS-*Gad1*^RNAi^ stocks were acquired from the Vienna Drosophila Resource Center (stock #32344GD and #330039; Dietzl et al., 2007), and the Synaptic Tagging with Recombination (STaR) effector line (w^-^; 20xUAS-RSR.PEST, 79C23S-RSRT-STOP-RSRT-smGFP_V5-2A-LexA/cyo; Peng et al., 2018) was shared by Orkun Akin (UCLA). nSyb-GRASP stocks (w*; P{w[+mC]=lexAop-nSyb-spGFP1-10}2, P{w[+mC]=UAS-CD4-spGFP11}2; MKRS/TM6B and w[*]; P{w[+mC]=UAS-nSyb-spGFP1-10}2, P{w[+mC]=lexAop-CD4-spGFP11}2/CyO; (Macpherson et al., 2015)), UAS-CD8::GFP (Pfeiffer et al., 2010), LexAOP-mCD4::RFP (Pfeiffer et al., 2010), UAS-CsChrimson::mCherry (Klapoetke et al., 2014), w[1118]; P{y[+t7.7] w[+mC]=R21C05-p65.AD}attP40; P{y[+t7.7] w[+mC]=R28E01-GAL4.DBD}attP2 (Namiki et al., 2018), and Trans-Tango (y[1] w[*] P{y[+t7.7] w[+mC]=UAS-myrGFP.QUAS-mtdTomato-3xHA}su(Hw)attP8; P{y[+t7.7] w[+mC]=trans-Tango}attP40) were provided by BDSC (stock #64314, #64315, #32186, #32229, #82181, #86737, #77124).

### Method details

#### Sleep

Sleep was measured as previously described (Shaw et al., 2002). Briefly, 3- to 7-d-old female flies were individually loaded into 65-mm-long glass tubes and inserted into *Drosophila* activity monitors (Trikinetics). Periods of inactivity lasting at least 5 min were classified as sleep. Sleep deprivation occurred mechanically via the Sleep Nullifying Apparatus (Shaw et al., 2002). Trikinetics activity records were analyzed for sleep using Visual Basic scripts (Shaw et al., 2002) in Microsoft Excel or the Sleep and Circadian Analysis MATLAB Program scripts (Donelson et al., 2012; Wiggin et al., 2020) in MATLAB (MathWorks). Multibeam monitoring experiments used MB5 monitors (Trikinetics) to record fly movements. For thermogenetic stimulations, flies were shifted from 25°C to either 31°C for daytime experiments or 30°C for nighttime experiments. The baseline temperature was maintained at 25°C to match the standard fly rearing conditions of the laboratory, and because physiological characterizations of dTrpA1 gating suggest minimal conductance at 25°C (Hamada et al., 2008; Luo et al., 2017). For optogenetic stimulation experiments, 6 h of constant illumination was delivered using an array of 225 LEDs with a total output of 1620 lux at 630 nm (model #8846671082812102, HQRP). Flies used in optogenetic experiments were reared in constant darkness following eclosion and fed standard fly media supplemented with either 0.125 mm all-trans retinal or vehicle control media (standard fly media with 0.625% EtOH) for at least 48 h before experiments. Optogenetic experiments were conducted using multibeam activity monitors (MB5, Trikinetics) to permit uniform illumination across each fly tube.

#### Arousability

Arousability was tested by attaching Trikinetics activity monitors to microplate adapters on vortexers (VWR). Vibration force intensities were measured using Vibration 3.83 (Diffraction Limited Design). Arousal tests used a 0.5 × *g* stimulation of 2 or 10 s duration. Flies that registered zero activity counts during the 5 min immediately before vibration were classified as asleep; previously sleeping flies that exhibited at least one beam break during the minute of stimulation or 1 min after stimulation were scored as awakened.

#### Antennal injury

Female flies were loaded into activity monitors at ∼4–7 d after eclosion, then permitted 1–2 d of baseline sleep. Antennae were bilaterally transected using fine forceps under CO_2_ anesthesia, then flies were returned to their tubes in activity monitors for recovery. Uninjured control siblings received matching CO_2_ concentration and exposure time.

#### Immunohistochemistry and confocal microscopy

*Drosophila* brains were dissected in PBS (1.86 mm NaH_2_PO_4_, 8.41 mm Na_2_HPO_4_, 175 mm NaCl; catalog #P4417, Sigma-Aldrich) and fixed in 4% (w/v) paraformaldehyde (catalog #15710-S, Electron Microscopy) in PBS for 30–45 min on ice. For GFP and RFP immunostaining, brains were incubated in primary antibody [1:1000; chicken anti-GFP; catalog #A10262, Thermo Fisher Scientific (RRID:AB_2534023); 1:1000; rabbit anti-DsRed; catalog #632496, Takara Bio (RRID:AB_10013483)] overnight followed by secondary antibody (1:1000; anti-chicken conjugated to Alexa Fluor 488; catalog #A11039, Thermo Fisher Scientific (RRID:AB_142924); 1:1000; anti-rabbit conjugated to Alexa Fluor 546, catalog #A11010, Thermo Fisher Scientific (RRID:AB_2534077)] for ∼24 h. Immunostaining for V5 used a 48 h incubation period in 1:400 mouse anti-V5 conjugated with DyLight550 (catalog #MCA1360GA, BIO-RAD; RRID:AB_567249). For GFP Reconstitution Across Synaptic Partners (GRASP) experiments, brains were incubated in primary antibodies [1:50; mouse anti-GFP; catalog #G6539-100UL, Sigma-Aldrich (RRID:AB_259941); 1:20; rat anti-N-cad; catalog #DN-EX #8, DSHB (RRID:AB_2314331)] for ∼48 h, followed by incubation in secondary antibodies [1:1000; anti-mouse conjugated to Alexa Fluor 488; catalog #A11001, Thermo Fisher Scientific (RRID:AB_2534069); 1:1000 anti-rat conjugated to Alexa Fluor 647; catalog #112–605-071, Jackson ImmunoResearch (RRID:AB_2338400)] for 48 h. Brains stained for GABA were incubated in primary antibody (1:500; rabbit anti-GABA; catalog #A2052-25UL, Sigma-Aldrich; RRID:AB_477652) overnight, then overnight in secondary antibody (1:1000; anti-rabbit conjugated with Alexa Fluor 546; catalog #A11010, Thermo Fisher Scientific; RRID:AB_2534077).

Trans-Tango flies were reared at 18°C for 15–21 d posteclosion, at which point they were dissected and fixed as described above, incubated in primary antibodies (as described above for GFP, RFP, and N-cad) overnight, followed by incubation in secondary antibodies (as described above for GFP, RFP, and N-cad) for 24 h.

All brains were mounted in Vectashield (catalog #H-1000, Vector Laboratories) and imaged on a Zeiss LSM 880 confocal microscope. All image processing was completed using Fiji (Schindelin et al., 2012). For large z*-*stacks, bright debris above or below the brain was manually erased to clearly visualize neuronal fluorescence. Quantification of STaR signal in ring neurons used a sum slices projection through the ellipsoid body and a background subtraction, followed by outlining the ring neurons and measuring area and mean anti-V5 intensity. To quantify anti-GABA strength in ER3m cell bodies, we first performed a background subtraction, then outlined each GFP-positive soma individually in the central *z*-slice corresponding to its maximal diameter and measured mean anti-GABA intensity.

Staining and mounting for genetic driver expression patterns in frontal and dorsal mount images (see Figs. 2, 6, 12, 13) were conducted as previously described (Omoto et al., 2017, 2018).

### Experimental design and statistical analysis

Each experiment included simultaneous data collection from age-matched experimental and control genotypes. Heterozygous controls were progeny of crosses between parental lines and Canton-S wild-type flies. All behavioral experiments were repeated in at least two independent experimental replicates.

Data were analyzed in Prism 9 (GraphPad). Group means were compared using two-tailed *t* tests or one- or two-way ANOVAs, with repeated measures where appropriate, followed by planned pairwise comparisons with multiple-comparisons tests as described in figure legends. Unless otherwise noted, asterisks indicate significant differences between experimental genotypes and all relevant genetic controls. Sample sizes for each experiment are depicted in each figure panel or in the appropriate figure legend. All group averages shown in data panels depict mean ± SEM (Table 1).

**Table 1:** Statistical table

Structure	Test	Result
1*A* – No assumptions	Two-way ANOVA	Genetic driver-by-TrpA1 interaction: *F*_(26,1730)_ = 22.21, *p* < 0.0001, *n* = 29–64 flies/genotype ; *p < 0.05 by Šídák’s pairwise comparison
1*B* – No assumptions	Two-way ANOVA	Genetic driver-by-TrpA1 interaction: *F*_(26,1888)_ = 20.05, *p* < 0.0001, *n* = 28–48 flies/genotype; *p < 0.05 by Šídák’s pairwise comparison
2*B* – No assumptions	Two-way repeated-measures ANOVA	Genotype-by-time interaction: *F*_(46,2875)_ = 33.26, *p* < 0.0001, *n* = 32–64 flies/group
2*D* – No assumptions	Two-way repeated-measures ANOVA	Genotype-by-time interaction: *F*_(8,668)_ = 71.81, *p* < 0.0001, *n* = 59–93 flies/group
2*E* - No assumptions	Two-way repeated-measures ANOVA	Genotype-by-time interaction: *F*_(8,668)_ = 4.593, *p* < 0.0001, *n* = 59-93 flies/group
2*F* – No assumptions	Two-way repeated-measures ANOVA	Genotype-by-time interaction: *F*_(8,665)_ = 75.76, *p* < 0.0001, *n* = 58–93
2G – No assumptions	Two-way repeated-measures ANOVA	Genotype-by-time interaction: *F*_(8,665)_ = 4.686, *p* < 0.0001, *n* = 58-93
2*H* – No assumptions	Two-way repeated-measures ANOVA	Genotype-by-time interaction: *F*_(8,664)_ = 14.99, *p* < 0.0001, *n* = 59-93 flies/group
3*A* – No assumptions	Two-way repeated-measures ANOVA	Genotype-by-time interaction: *F*_(46,2139)_ = 20.28, *p* < 0.0001, *n* = 32 flies/group
3*B* - No assumptions	Two-way repeated-measures ANOVA	Genotype-by-time interaction: *F*_(10,321)_ = 17.31, *p* < 0.0001, *n* = 15–51 flies/group; *Holm–Šídák’s multiple-comparisons; *p* < 0.0001 when compared with both UAS-*TrpA1*/+ and Split-Gal4/+ genetic controls
3*F* (left) – Nonparametric	Mann–Whitney test	Mann–Whitney *U* = 419, *p* = 0.54
3*F* (right) – Normal distribution	Unpaired *t* test	*t* = 0.7358, df = 59, *p* = 0.4648
4*B* – No assumption	Two-way repeated-measures ANOVA	Genotype-by-time interaction: *F*_(23,5543)_ = 7.27, *p* < 0.0001, *n* = 118–125 flies/group
4*F* – No assumption	Two-way repeated-measures ANOVA	Genotype-by-time interaction: *F*_(13,2875)_ = 15.51, *p* < 0.0001, *n* = 63–64 flies/group
4*G* – No assumption	Two-way repeated-measures ANOVA	Genotype-by-time interaction: *F*_(2,341)_ = 108.1, *p* < 0.0001, *n* = 64–160 flies/group
4*H* – No assumption	Two-way repeated-measures ANOVA	Genotype-by-time interaction: *F*_(3,123)_ = 79.5, *p* < 0.0001, *n* = 31–32 flies/group
5*F* - No assumption	Two-way repeated-measures ANOVA	Genotype-by-time interaction: *F*_(1,101)_ = 2.803, *p* = 0.097, *n* = 45–58 flies/group
5*G* – No assumption	Two-way repeated-measures ANOVA	Genotype-by-time interaction: *F*_(46,12374)_ = 15.59, *p* < 0.0001, *n* = 158–192
5*H* – No assumption	Two-way repeated-measures ANOVA	Genotype-by-time interaction: *F*_(46,10097)_ = 18.33, *p* < 0.0001, *n* = 95–192
5*I* – No assumption	Two-way repeated-measures ANOVA	Genotype-by-time interaction: *F*_(46,2093)_ = 3.908, *p* < 0.0001, *n* = 31–32
5*J* - No assumption	Two-way repeated-measures ANOVA	Genotype-by-time interaction: *F*_(46,2093)_ = 3.027, *p* < 0.0001, *n* = 30–32
6*B* - No assumption	Two-way repeated-measures ANOVA	Genotype-by-time interaction: *F*_(46,3496)_ = 29.46, *p* < 0.0001, *n* = 48–59 flies/group
6*D* - No assumption	Two-way repeated-measures ANOVA	Genotype-by-time interaction: *F*_(8,478)_ = 78.15, *p* < 0.0001, *n* = 30–88 flies/group
6*I* - No assumption	Two-way repeated-measures ANOVA	Genotype-by-time interaction: *F*_(8,478)_ = 21.99, *p* < 0.0001, *n* = 30–88 flies/group
6*J* – No assumption	Two-way repeated-measures ANOVA	Genotype-by-time interaction: *F*_(8,478)_ = 23.90, *p* < 0.0001, *n* = 30–88 flies/group
6*K* – No assumption	Two-way repeated-measures ANOVA	Genotype-by-time interaction: *F*_(8,478)_ = 23.77, *p* < 0.0001, *n* = 30–88 flies/group
6*L* - No assumption	Two-way repeated-measures ANOVA	Genotype-by-time interaction: *F*_(8,450)_ = 2.895, *p* = 0.0037, *n* = 31–88 flies/group
7*A* – No assumption	One-way repeated-measures ANOVA	Main effects for genotype: 2 s trials: *F*_(4,37)_ = 20.98, *p* < 0.0001, *n* = 5–12 trials/group; 10 s trials: *t* test *p* = 0.064, *t* = 2.024, *n* = 6–9 trials/group
7*B* – No assumption	Two-way repeated-measures ANOVA	Genotype-by-time interaction: *F*_(46,2070)_ = 16.58, *p* < 0.0001, *n* = 30–32 flies/group
7*C* – No assumption	Two-way repeated-measures ANOVA	Genotype-by-time interaction: *F*_(8,270)_ = 46.03, *p* < 0.0001, *n* = 16–48 flies/group
7*D* – No assumption	Two-way repeated-measures ANOVA	Group-by-time interaction: *F*_(235,13677)_ = 7.58, *p* < 0.0001, *n* = 32–61 flies/group
7*E* - No assumption	Two-way repeated-measures ANOVA	Group-by-time interaction: *R28E01-*Gal4: *F*_(5,288)_ = 34.09, *p* < 0.0001, *n* = 32–61 flies/group; *R47D08*-Gal4: *F*_(5,312)_ = 16.02, *p* < 0.0001, *n* = 32–64 flies/group
7*G* (left) - Parametric	Unpaired *t* test	*t* = 3.087, df = 60, *p* = 0.0031
7*G* (right) - Parametric	Unpaired *t* test	*t* = 0.5558, df = 60, *p* = 0.5804
8*D* – No assumptions	Two-way repeated-measures ANOVA	Genotype-by-time interaction: *F*_(2,374)_ = 18.03, *p* < 0.0001, *n* = 93–96 flies/group
8*E* – No assumptions	Two-way repeated-measures ANOVA	Genotype-by-time interaction: *F*_(46,2024)_ = 3.553, *p* < 0.0001, *n* = 29–32
8*F* – No assumptions	Two-way repeated-measures ANOVA	Genotype-by-time interaction: *F*_(2,88)_ = 9.78, *p* < 0.0001, *n* = 29–32
8*H* (left) – No assumptions	Two-way repeated-measures ANOVA	Genotype-by-time interaction: *F*_(46,5842)_ = 43.09, *p* < 0001, *n* = 44–125 flies/group
8*H* (right) – Nonparametric	Kruskal–Wallis test	Kruskal–Wallis statistic = 96.35, *p* < 0.0001, *n* = 44–125 flies/group
10*D* – Nonparametric	Kruskal–Wallis test	Kruskal–Wallis statistic = 24.24, *p* < 0.0001, *n* = 102–223 cells/group from 5–9 flies/group
10*E* - No assumptions	Two-way repeated-measures ANOVA	Genotype-by-time interaction: *F*_(46,9936)_ = 35.72, *p* < 0.0001, *n* = 92–188
10*F* - No assumptions	Two-way repeated-measures ANOVA	Genotype-by-time interaction: *F*_(46,12305)_ = 32.87, *p* < 0.0001, *n* = 159–191
10*G* - No assumptions	Two-way repeated-measures ANOVA	Genotype-by-time interaction: *F*_(46,3887)_ = 8.543, *p* < 0.0001, *n* = 59–64
10*H* - No assumptions	Two-way repeated-measures ANOVA	Genotype-by-time interaction: *F*_(46,4301)_ = 15.28, *p* < 0.0001, *n* = 63-64
10*J* – Nonparametric	Kruskal-Wallis test	Kruskal–Wallis statistic = 77.49, *n* = 31-32; *p < 0.05 between experimental genotype and both genetic controls using; Dunn's multiple-comparisons test
10*K* – Parametric	One-way ANOVA	Main effect of genotype: *F*_(5,173)_ = 18.93, *p* < 0.0001, *n* = 24–32 flies/group ; *p < 0.05 between experimental genotype and both genetic controls using; Šídák's multiple-comparisons test
11*B* – No assumptions	Two-way repeated-measures ANOVA	Genotype-by-time interaction: *F*_(46,4324)_ = 1.199, *p* = 0.1687, *n* = 63–64 flies/group
11*F* – No assumptions	Two-way repeated-measures ANOVA	Genotype-by-time interaction: *F*_(8,365)_ = 1.828, *p* = 0.0706, *n* = 30–63 flies/group
11*G* – No assumptions	Two-way repeated-measures ANOVA	Group-by-time interaction: *F*_(115,3473)_ = 6.154, *p* < 0.0001, *n* = 16–32 flies/group
11*H* – No assumptions	Two-way repeated-measures ANOVA	Group-by-time interaction: *F*_(17,413)_ = 23.03, *p* < 0.0001, *n* = 16–32 flies/group

### Data availability

The published article includes all datasets generated during this study. This study did not generate any novel code. Further information and requests for resources and reagents should be directed to and will be fulfilled by author J.M.D., the lead contact. This study did not generate new unique reagents.

## Results

### EB mini-screen for sleep-regulatory ER neurons

**Figure 1.**
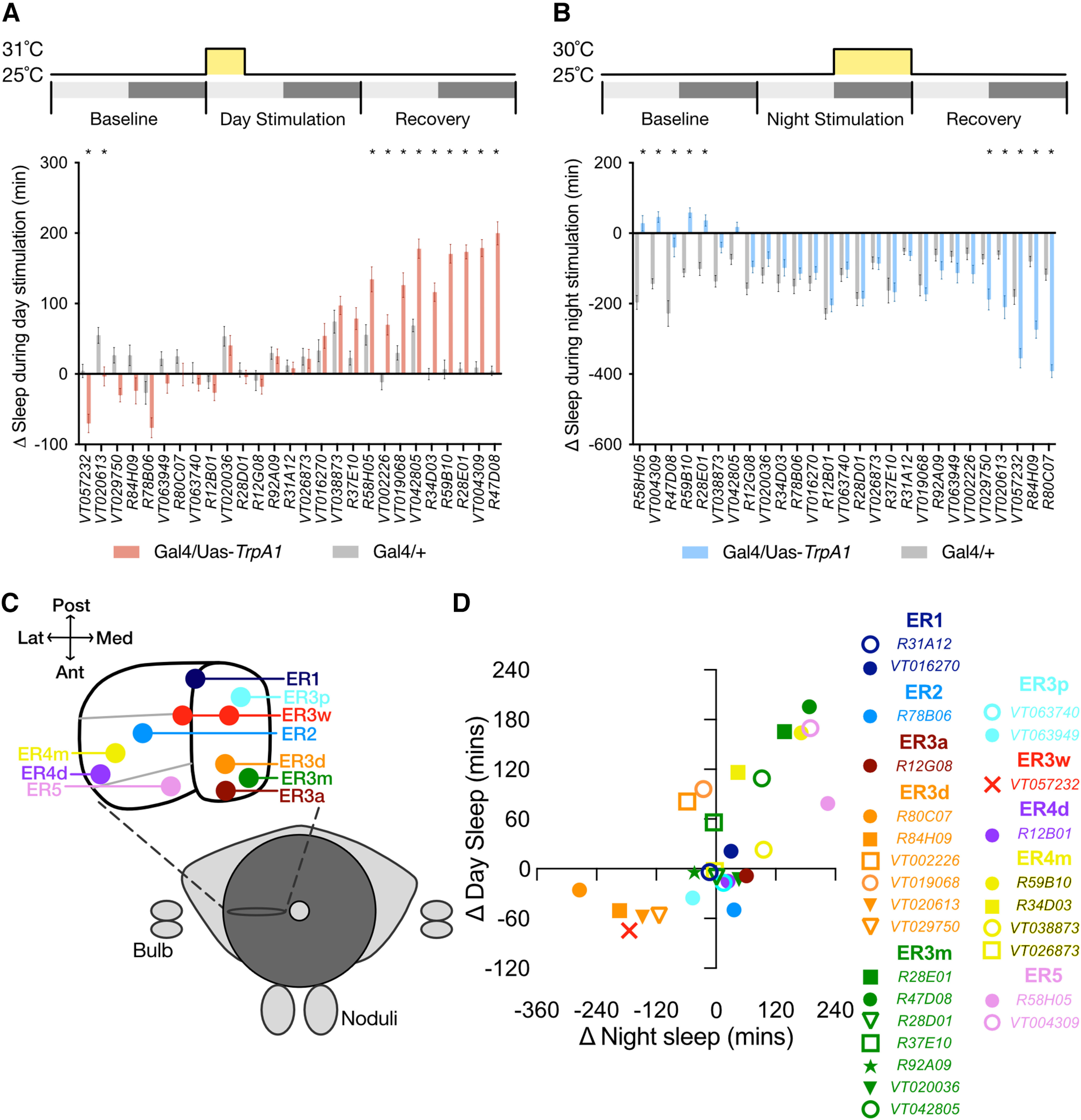
Characterizing sleep regulatory roles for distinct classes of Ring neurons. ***A***, ***B***, Sleep results from targeted mini-screen for heat activation of Gal4 genetic drivers that label individual subtypes of EB Ring neurons. Change in sleep for each individual fly between heat exposure from ZT0 to ZT6 (***A***; 31°C) or from ZT12 to ZT0 (***B***; 30°C) compared with sleep from the same fly during the previous baseline day. Gray depicts Gal4/+ controls for each driver; red or blue represents Gal4>UAS-*TrpA1* experimental lines. Significant Driver-by-Presence of *TrpA1* interactions were found for both daytime (*F*_(26,1730)_ = 22.21, *p* < 0.0001, *n* = 29–64 flies/genotype) and nighttime (*F*_(26,1888)_ = 20.05, *p* < 0.0001, *n* = 28–48 flies/genotype) mini-screens. *Drivers with *p* < 0.05 by pairwise Šídák’s multiple-comparisons test. ***C***, Schematic of the *Drosophila* Central Complex, including a frontal cross section of the ellipsoid body illustrating projection regions for 10 distinct Ring neuron subtypes. Based on Omoto et al. (2018). ***D***, Scatter plot depicting the changes in sleep for each Gal4 driver included in the sleep mini-screen resulting from heat exposure during the night (*x*-axis) and day (*y*-axis). Day or night sleep effects were calculated by subtracting the Gal4/+ changes in sleep from the Gal4/*TrpA1* changes in sleep shown in ***A*** and ***B*** for each driver. Datapoints are color coded by the putative Ring neuron subtypes in which each Gal4 driver is primarily expressed.

Previous studies have identified neuron types within the *Drosophila* central complex that modulate sleep (Liu et al., 2016; Pimentel et al., 2016; Donlea et al., 2018; Ho et al., 2022). Whether additional circuitry also influences sleep-related signals in the EB, however, has not been clearly described. To examine the roles of other EB ring neurons in sleep regulation, we completed a targeted thermogenetic screen of genetic driver lines that drive expression in various ER neuron subclasses (Jenett et al., 2012; Tirian and Dickson, 2017; Omoto et al., 2018). Twenty-seven genetic driver lines that label different subclasses of ER neurons were each used to express the warm-sensitive cation channel *TrpA1* (Hamada et al., 2008). Experimental (Gal4/UAS-*TrpA1*) and genetic control flies (Gal4/+) for each driver were loaded into activity monitors for 1–2 d of baseline sleep, then were heated to 31°C either for 6 h from zeitgeber time 0 (ZT0) to ZT6 (Fig. 1*A*) or to 30°C for 12 h from ZT12 to ZT0 (Fig. 1*B*). Since sleep in wild-type flies is relatively low during the first few hours after ZT0 and high during the night, we hypothesized that the morning stimulation would enable us to preferentially identify sleep-promoting neurons while nighttime activation would identify arousing ER neuron subclasses. At the light microscopy level, ER neurons can be divided into 11 morphologically distinct subclasses based on the locations of their dendritic arbors in the bulb or lateral accessory lobe and of their axonal projections into the EB (Fig. 1*C*, schematic; Materials and Methods, see detailed description; Omoto et al., 2018). Notably, the 11 subclasses can be further divided into 22 individual types with the inclusion of connectivity criteria from the hemibrain connectomics dataset (Hulse et al., 2021). *TrpA1* activation of many ER neuron subclasses, including ER1, ER2, ER3a, ER3p, and ER4d, elicited little change in sleep time during either heat protocol. Activation of ER5 neurons during either the day or night resulted in acute increases in sleep. Previous studies have identified sleep-promoting effects of ER5 stimulation (Liu et al., 2016; Ho et al., 2022), but an additional report attributed phenotypes of some ER5 drivers to peripheral expression within wake-promoting sensory neurons located in the legs (Satterfield et al., 2022). From our initial screen results, we selected two strongly sleep-promoting driver lines, *R28E01-*Gal4 and *R47D08*-Gal4 (Jenett et al., 2012), and two strongly wake-promoting driver lines, *R80C07*-Gal4 and *R84H09*-Gal4, for further analysis (Fig. 1*C*,*D*). These drivers were selected by the strength of their mini-screen phenotypes, the morphologic similarities of the ring neurons within each driver pair, and because they did not label ER5 neurons, which have already been linked with sleep regulation.

**Figure 2.**
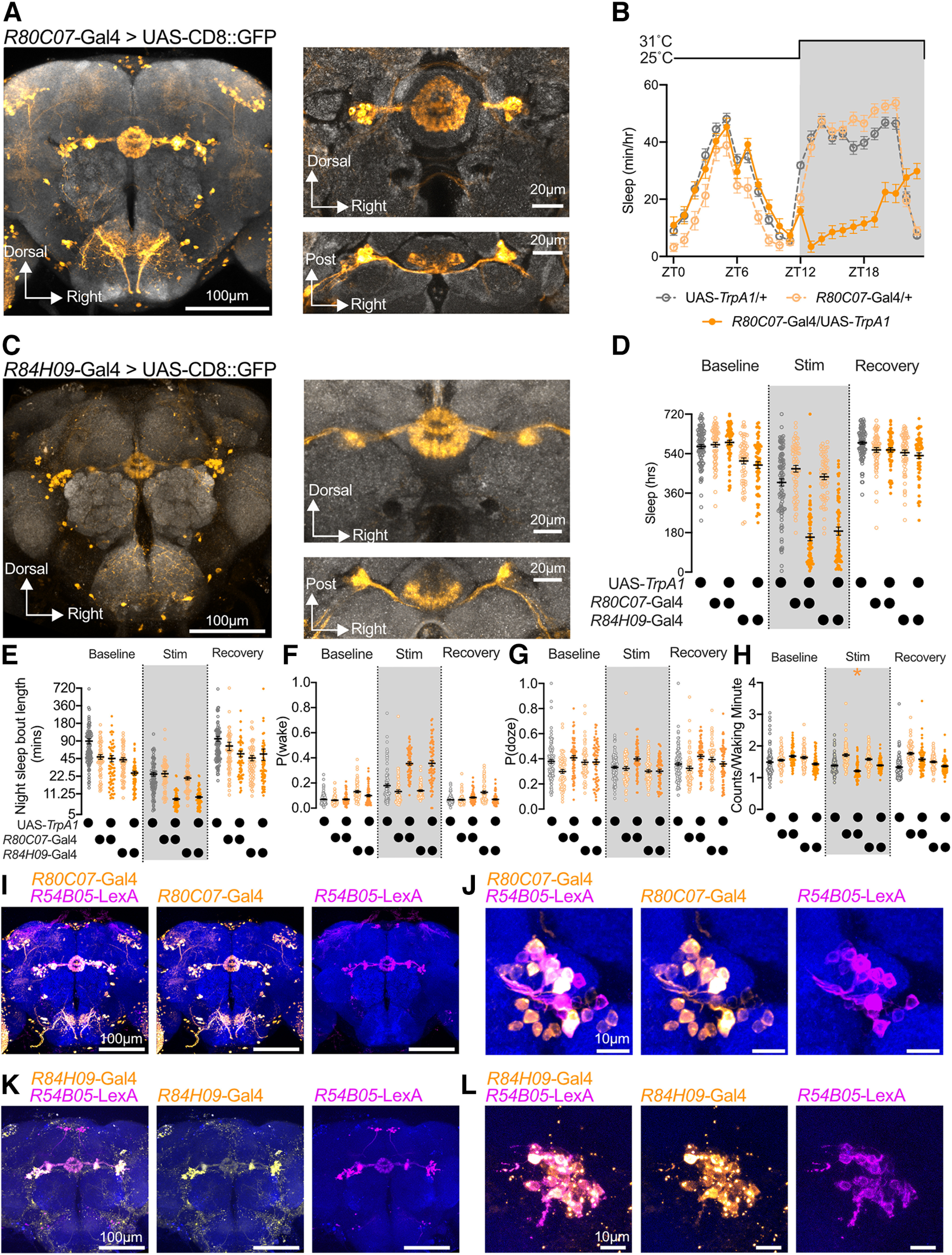
Activation of two ER3d-expressing drivers promotes waking via reduced sleep maintenance. ***A***, Expression of CD8::GFP under the control of *R80C07-*Gal4 labels ER3d ring neurons along with cells in the SEZ and dorsal protocerebrum. Left, *z*-Projection of central brain confocal scan. Right, Central complex expression of *R80C07*-Gal4 shown as projections of higher magnification confocal scans from frontal (top) or dorsal (bottom) angles. ***B***, Hourly sleep time course of *R80C07*-Gal4/UAS-*TrpA1* experimental flies (dark orange) compared with *R80C07*-Gal4/+ (light orange) and UAS-*TrpA1*/+ (gray) controls during a 12 h overnight heat exposure (30°C; yellow shading) from ZT12 to ZT24. Two-way repeated-measures ANOVA finds a significant time-by-genotype interaction (*F*_(46,2875)_ = 33.26, *p* < 0.0001, *n* = 32–64 flies/group). ***C***, CD8::GFP driven with *R84H09*-Gal4 is highly specific for ER3d neurons. Left, Central brain confocal scan; central complex expression depicted on right from frontal (top) and dorsal (bottom) views. ***D***, Nighttime sleep is decreased in *R80C07*-Gal4>UAS-*TrpA1* and *R84H09*-Gal4>UAS-*TrpA1* flies (dark orange) compared with Gal4/+ (light orange) and UAS-*TrpA1*/+ (gray) controls. Two-way repeated-measures ANOVA finds a significant day-by-genotype interaction (*F*_(8,668)_ = 71.81, *p* < 0.0001, *n* = 59–93 flies/group). ***E***, The mean duration of nighttime sleep bouts is decreased in *R80C07-*Gal4>UAS-*TrpA1* and *R84H09*-Gal4>UAS-*TrpA1* experimental flies (dark orange) during heat stimulation compared with genetic controls (light orange and gray). Two-way repeated-measures ANOVA finds a significant day-by-genotype interaction (*F*_(8,668)_ = 4.593, *p* < 0.0001, *n* = 59–93 flies/group). ***F***, ***G***, P(wake) (***F***), P(doze) (***G***) for ER3d activation; experimental flies (dark orange) show increased P(wake) during overnight heat exposure compared with genetic controls (Gal4/+ groups in light orange and UAS-*TrpA1*/+ in gray), while no decrease in P(doze) was detected. Two-way repeated-measures ANOVA finds a significant day-by-genotype interaction for P(wake) (*F*_(8,665)_ = 75.76, *p* < 0.0001, *n* = 58–93) and P(doze) (*F*_(8,665)_ = 4.686, *p* < 0.0001, *n* = 58–93). (***H***) Waking activity (counts/waking minute) in experimental flies (orange) compared with genetic controls (Gal4/+ in light orange; UAS-TrpA1/+ in gray). Two-way repeated-measures ANOVA finds a significant day-by-genotype interaction (*F*_(8,664)_ = 14.99, *p* < 0.0001, *n* = 59–93 flies/group). *p < 0.05 compared with Gal4/+ and UAS-*TrpA1*/+ controls by Tukey’s multiple-comparison test). ***I***, ***J***, *R54B05*-LexA (magenta) labels a large subset of ER3d neurons that also express *R80C07*-Gal4 (orange). Central brain (***I***); higher-magnification projections of ER3d soma (***J***). ***K***, ***L***, Nearly all ER3d neurons that are driven by *R84H09*-Gal4 (orange) also express *R54B05*-LexA (magenta). Central brain projection (***K***), higher magnification of ER3d cell bodies (***L***). For whole-brain *z*-stacks used in Figure 2, *A* and *C*, bright autofluorescent debris present in slices above or below the brain were manually erased to prevent blockade of neuronal fluorescence on maximum intensity *z*-projection.

### Wake-promoting genetic drivers label coincident ER3d neurons

**Figure 3.**
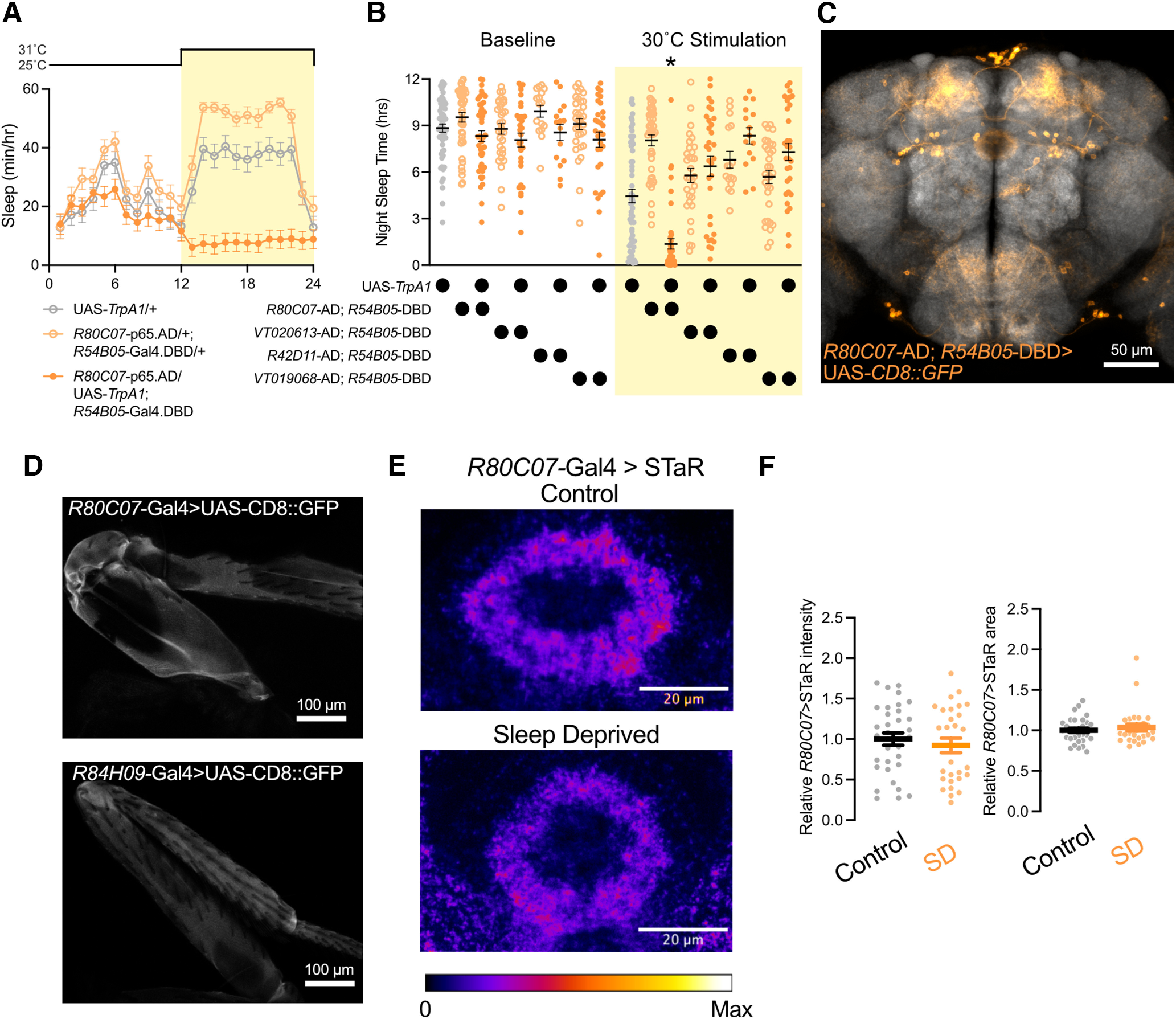
Characterization of ER3d driver phenotypes and ER3d plasticity. ***A***, Hourly sleep time course during overnight heat stimulation (ZT12–ZT0; 30°C) for *R80C07*-p65.AD; *R54B05-*Gal4.DBD > UAS-*Trpa1* flies (filled orange circles) and for genetic controls (UAS-TrpA1/+ in open gray circles; *R80C07*-p65.AD; *R54B05*-Gal4.DBD/+ in open orange circles). Two-way repeated-measures ANOVA finds a significant time-by-genotype interaction (*F*_(46,2139)_ = 20.28, *p* < 0.0001, *n* = 32 flies/group). ***B***, Overnight sleep time (ZT12–ZT0) for baseline (left) and heat stimulation night (right) for experimental flies using Split-Gal4s to drive UAS-*TrpA1* (filled orange circles) and genetic controls (open gray circles for UAS-*TrpA1*/+ and open orange circles for Split-Gal4/+). Sleep was significantly reduced in *R80C07*-p65.AD; *R54B05-*Gal4.DBD > UAS-*Trpa1* flies relative to both control genotypes, but not with other Split-Gal4 combinations. Two-way repeated-measures detects a significant night-by-genotype interaction (*F*_(10,321)_ = 17.31, *p* < 0.0001, *n* = 15–51 flies/group). *Holm–Šídák’s multiple-comparisons test, *p* < 0.0001 when compared with both UAS-*TrpA1*/+ and Split-Gal4/+ genetic controls. ***C***, Confocal projection of central brain from *R80C07*-p65.AD; *R54B05-*Gal4.DBD > UAS-CD8::GFP fly. ***D***, Confocal micrographs of legs from *R80C07*>CD8::GFP (top) and *R84H09*>CD8::GFP (bottom). ***E***, Example images from control (top) and sleep-deprived (bottom) brains labeling presynaptic active zones with BRP::smFP_V5 in ER3d neurons using *R80C07*-Gal4. ***F***, Mean relative intensity (left) and cross-sectional area (right) of presynaptic BRP labeled by STaR expression in ER3d neurons using *R80C07*-Gal4. Sleep deprivation had no significant effect on relative STaR intensity (Mann–Whitney test, *p* = 0.5412) or area (two-tailed *t* test, *p* = 0.4648, *n* = 28–33 brains/group).

Our ring neuron mini-screen shown in Figure 1 indicated that two Gal4 drivers that primarily label ER3d neurons, *R80C07-*Gal4 (Fig. 2*A*) and *R84H09*-Gal4 (Fig. 2*C*), promote wakefulness on overnight activation with *TrpA1* (Fig. 2*B*,*D*). The reduction in sleep that occurs during activation of *R80C07*-Gal4 or *R84H09-*Gal4 coincides with a fragmentation in sleep bout length (Fig. 2*E*). Sleep loss during ER3d activation with *R80C07*-Gal4 or *R84H09*-Gal4 can be attributed to a reduction in the persistence of sleep bouts. The probability that a sleeping fly will awaken, P(wake), is significantly elevated with ER3d activation compared with genetic controls (Fig. 2*F*), while the probability of sleep initiation, P(doze), remains unaffected by ER3d stimulation (Fig. 2*G*). ER3d stimulation using *R80C07-*Gal4 also caused a modest decrease in activity counts per waking minute (Fig. 2*H*), suggesting that ER3d neurons do not drive hyperarousal. To confirm that both wake-promoting drivers express in the same population of ER3d neurons, we coexpressed each Gal4 with the orthogonal ER3d driver *R54B05-*LexA. While *R54B05-*LexA labels only a subset of the ER neurons labeled by *R80C07*-Gal4 and *R84H09*-Gal4, these results confirm that both wake-promoting drivers are expressed in an overlapping ER3d population (Fig. 2*I–L*). We tested whether *R54B05*-positive cells within the *R80C07-*Gal4 expression pattern increased waking on activation by thermogenetically stimulating *TrpA1* under the control of *R80C07*-p65.AD; *R54B05*-Gal4.DBD. Activating this Split-Gal4 driver was sufficient to suppress sleep overnight (Fig. 3*A*), and other Split-Gal4 combinations for ER3d-expressing hemidrivers did not elicit similar changes in waking (Fig. 3*B*). While the *R80C07*-p65.AD; *R54B05*-Gal4.DBD does express in ER3d neurons, it also labels other neurons in the central brain (Fig. 3*C*). To test whether arousal-promoting leg sensory neurons might be labeled by *R80C07*-Gal4 or *R84H09*-Gal4 (Satterfield et al., 2022), we imaged legs from *R80C07*-Gal4>UAS-CD8::GFP and *R84H09*-Gal4>UAS-CD8::GFP; we detected autofluorescence from cuticle, but found no evidence for GFP-positive leg neurons (Fig. 3*D*). Unlike in ER5 and ER3m neurons (Fig. 7*F*, below), STaR labeling (Chen et al., 2014; Peng et al., 2018) shows no significant change in the pre-synaptic active zone abundance of ER3d neurons after overnight sleep loss (Fig. 3*E*,*F*). This is consistent with previous results indicating that the presynaptic scaffolding protein Bruchpilot (BRP) may scale differently between ER neuron subclasses during sleep deprivation (Liu et al., 2016).

### Activation of GABAergic ER3d neurons increases wakefulness

**Figure 4.**
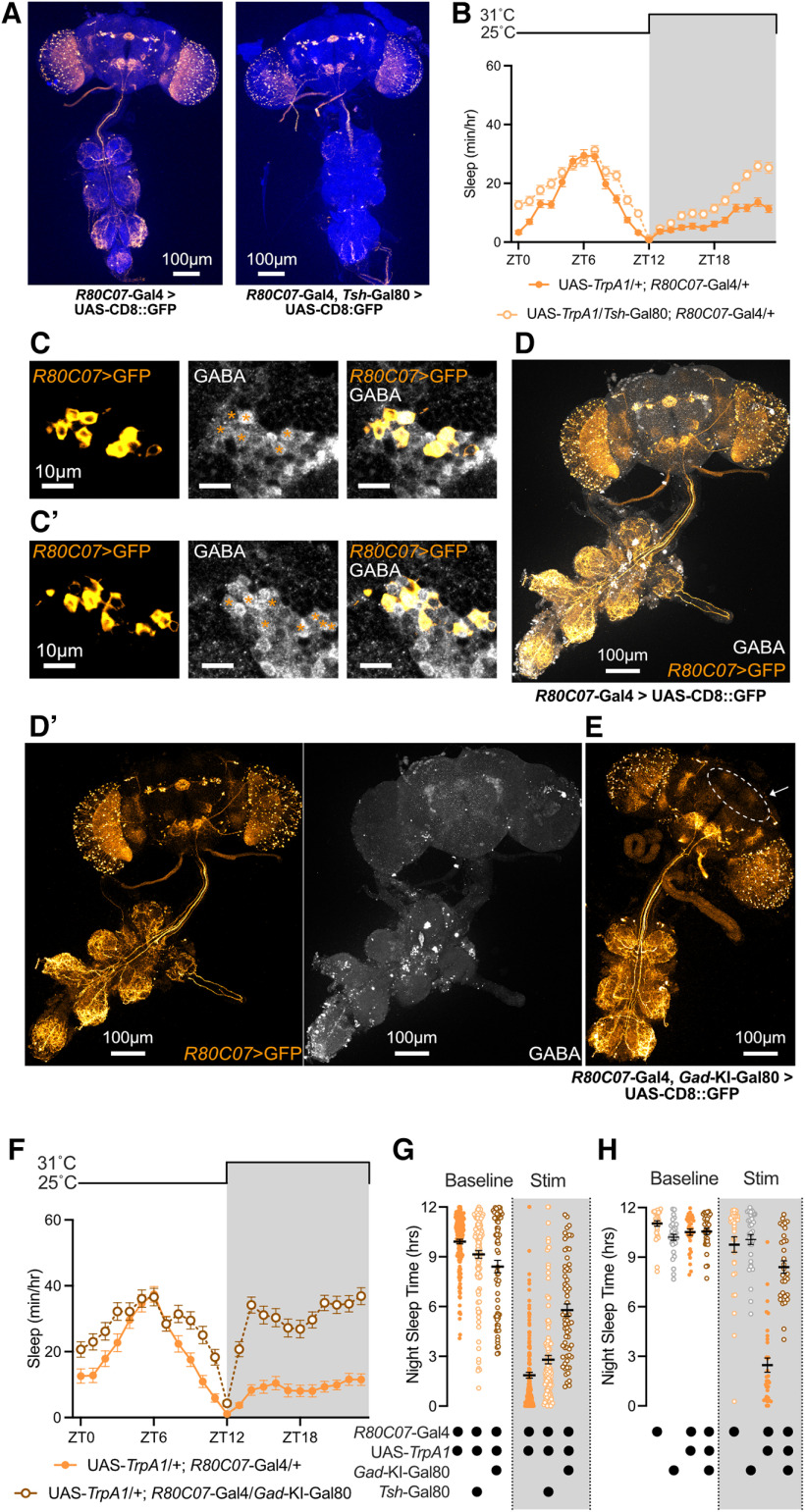
*R80C07-*positive ER3d neurons overlap with GABA immunostaining and suppress sleep. ***A***, CD8::GFP expression in the whole brain and VNC under the control of *R80C07-*Gal4 (left) and *R80C07*-Gal4 paired with *Tsh*-Gal80 (right). ***B***, Hourly sleep for UAS-*TrpA1*/+; *R80C07*-Gal4/+ (orange) and UAS-*TrpA1*/*Tsh-*Gal80; *R80C07*-Gal4/+ (light orange) while flies were housed at 25°C during ZT0–ZT12 and 31°C from ZT12 to ZT24. Two-way repeated-measures ANOVA finds a significant time-by-genotype interaction (*F*_(23,5543)_ = 7.27, *p* < 0.0001, *n* = 118–125 flies/group). ***C***, ER3d neurons labeled with *R80C07*-Gal4>UAS-CD8::GFP overlap with immunostaining for GABA. ***C***, ***C′***, Different confocal slices from an individual *z*-stack. Asterisks mark Gal4-labeled ER3d soma that express GABA. ***D***, Whole-brain and VNC expression of UAS-CD8::GFP driven by *R80C07*-Gal4 (orange) along with anti-GABA immunostaining (white). ***D*′**, Individual channels alone; *R80C07*-Gal4>UAS-CD8::GFP on left, and anti-GABA on right. ***E***, Expression pattern of UAS-CD8::GFP/*+*; *R80C07*-Gal4/*Gad1*-KI-Gal80 in the brain and VNC. We observed no GFP expression in the EB (outlined by dashed oval), indicating a loss of labeling in ER3d neurons. ***F***, Sleep time course for UAS-*TrpA1*/+; *R80C07*-Gal4/+ (orange) and UAS-*TrpA1*/*+*; *R80C07*-Gal4/*Gad1*-KI-Gal80 (brown). Flies were warmed to 30°C for ZT12–ZT24. Two-way repeated-measures ANOVA finds a significant time-by-genotype interaction (*F*_(13,2875)_ = 15.51, *p* < 0.0001, *n* = 63–64 flies/group). ***G***, ***H***, Nighttime sleep for groups shown in ***B*** and ***F*** during a baseline night at 25°C followed by a stimulation night during which flies were housed at 30°C. Two-way repeated-measures ANOVA finds a significant time-by-genotype interaction in ***G*** (*F*_(2,341)_ = 108.1, *p* < 0.0001, *n* = 64–160 flies/group) and in ***H*** (*F*_(3,123)_ = 79.5, *p* < 0.0001, *n* = 31–32 flies/group).

*R80C07*-Gal4 labels ER3d neurons, but also drives expression in other regions of the CNS, including the ventral nerve cord (VNC), optic lobes, subesophageal zone (SEZ), and dorsal protocerebrum (Fig. 4*A*, left). To minimize the effects of activating proprioceptive and motor circuits in the VNC, we combined *R80C07*-Gal4 with *Tsh*-Gal80 (Fig. 4*A*, right). The wake-promoting effects of activating *R80C07*-Gal4 were only modestly weakened when paired with *Tsh-*Gal80 (Fig. 4*B*,*G*), indicating that VNC neurons cannot account for arousal during *R80C07-*Gal4 stimulation. Next, we used immunostaining to find that ER3d neurons labeled by *R80C07*-Gal4 express inhibitory GABA (Fig. 4*C*), but little colocalization between GABA staining and *R80C07*-Gal4 is found outside of the ellipsoid body (Fig. 4*D*,*D*′).

**Figure 5.**
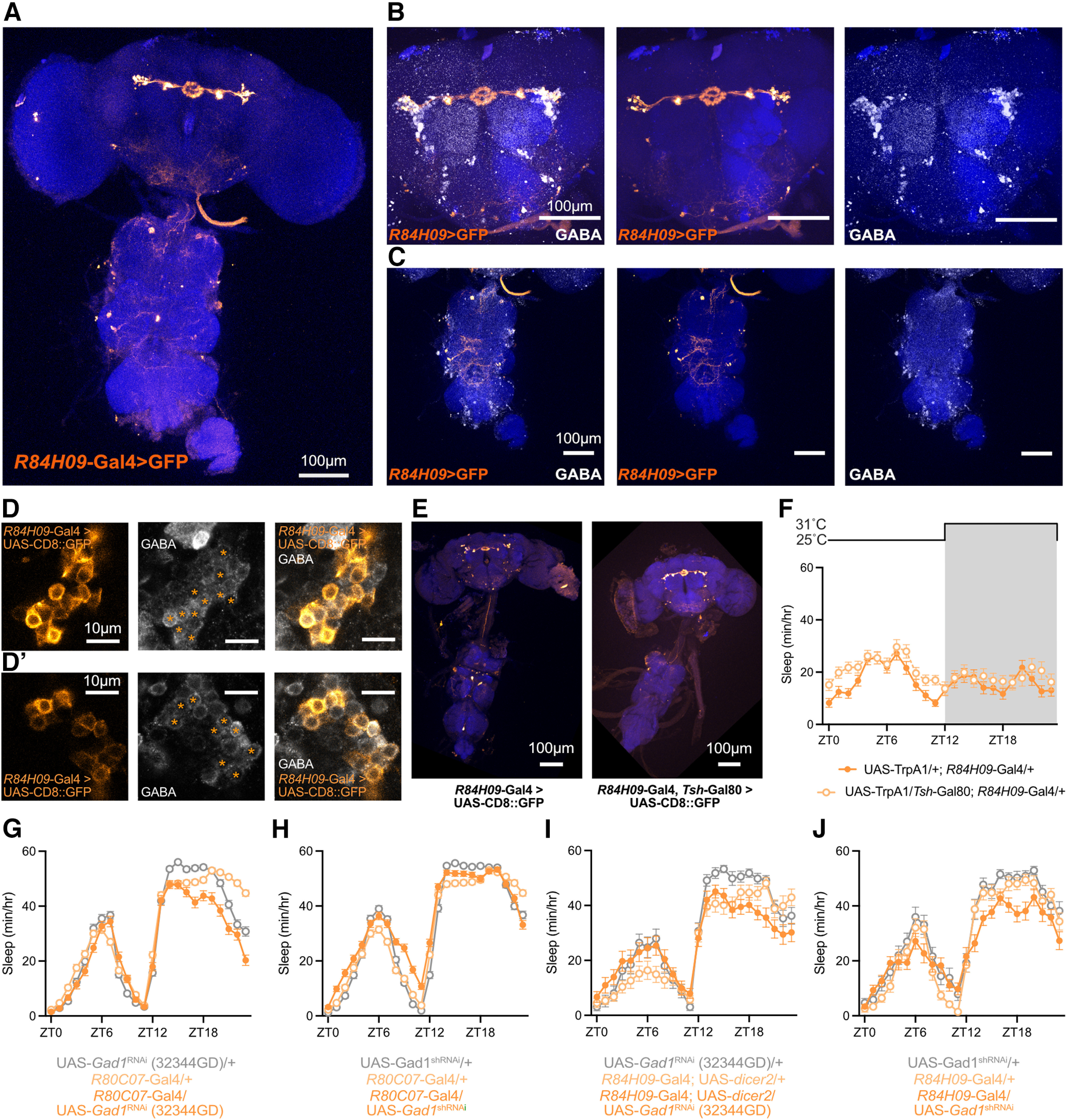
*R84H09*-positive ER3d neurons colocalize with GABA immunostaining. ***A***, Whole-brain and VNC labeling of *R84H09*-Gal4>UAS-CD8::GFP. ***B***, ***C***, Colocalization of *R84H09*-Gal4>UAS-CD8::GFP (Orange) and GABA immunostaining (white) in the central brain (***B***) and VNC (***C***) is specific for ER3d neurons. ***D***, ER3d neurons tagged by *R84H09*-Gal4>UAS-CD8::GFP costain for anti-GABA. ***D***, ***D′***, Independent confocal slices; asterisks represent *R84H09-*Gal4-positive soma that overlap with anti-GABA staining. ***E***, *z*-Projections through the whole brain and VNC of *R84H09*-Gal4>UAS-CD8::GFP and *R84H09*-Gal4, *Tsh-*Gal80>UAS-CD8::GFP flies. Image in right panel was rotated for visual presentation and a black background was extended to fill the rectangular panel. ***F***, Hourly sleep time course for UAS-*TrpA1*/+; *R84H09*-Gal4/+ (orange, filled circles) and UAS-*TrpA1*/*Tsh*-Gal80; *R84H09*-Gal4/+ (light orange, empty circles) while flies were housed at 25°C during ZT0–ZT12 and shifted to 31°C from ZT12 to ZT24. Two-way repeated-measures ANOVA found no significant effect of genotype (*F*_(1,101)_ = 2.803, *p* = 0.097, *n* = 45–58 flies/group). ***G–J***, Hourly sleep time courses for ER3d-driven knockdown of Gad1 expression using two RNAi lines. *R80C07*-Gal4 driven UAS-*Gad1*^RNAi^ (32344GD; ***G***) and UAS-*Gad1*^shRNA^ (***H***) on left; expression using *R84H09*-Gal4 on right [UAS-*Gad1*^RNAi^ (32344GD; ***I***); UAS- *Gad1*^shRNA^ (***J***)]. Two-way repeated-measures ANOVA finds a significant time-by-genotype interaction for ***G*** (*F*_(46,12374)_ = 15.59, *p* < 0.0001, *n* = 158–192), ***H*** (*F*_(46,10097)_ = 18.33, *p* < 0.0001, *n* = 95–192), ***I*** (*F*_(46,2093)_ = 3.908, *p* < 0.0001, *n* = 31–32), and ***J*** (*F*_(46,2093)_ = 3.027, *p* < 0.0001, *n* = 30–32).

To test whether activation of GABAergic ER3d cells is required for the wake-promoting effects of *R80C07-*Gal4 stimulation, we next used a *Gad1*-KI-Gal80 line in which Gal80 is inserted into the genomic *Gad1* locus using a previously described knock-in strategy (Deng et al., 2019). As shown in Figure 4*E*, *Gad1*-KI-Gal80 subtracted ER3d neurons from the expression pattern of *R80C07*-Gal4, but much of the other Gal4 expression from this driver remained unaffected. Combining the *Gad1*-KI-Gal80 repressor with *R80C07*-Gal4 suppressed arousal during thermogenetic stimulation to a greater degree than *Tsh*-Gal80 (Fig. 4*F–H*), suggesting that GABAergic ER3d cells drive the wake-promoting effects of *R80C07*-Gal4 activation. Similarly, ER3d neurons labeled with *R84H09*-Gal4 overlap with anti-GABA immunostaining (Fig. 5*A*–*D*). However, RNAi-mediated knockdown of Gad1 in ER3d neurons using *R80C07*-Gal4 or *R84H09*-Gal4 did not consistently alter daily sleep patterns (Fig. 5*G*–*J*), indicating that GABAergic signaling from ER3d cells may not be required for baseline sleep regulation or that RNAi mediated depletion of Gad1 may not sufficiently reduce GABA levels to drive a behavioral phenotype. Next, we tested whether ER3d neurons contribute to the wake-promoting effects of *R84H09*-Gal4 activation by subtracting VNC expression with *Tsh*-Gal80. The expression pattern of *R84H09*-Gal4 includes several cell bodies within the VNC (Fig. 5*A*,*C*), but the addition of *Tsh*-Gal80 removes those somata (Fig. 5*E*). Restricting expression of *R84H09*-Gal4 leaves strong expression in ER3d neurons with weaker labeling in only a handful of other neurons in the brain. We find that thermogenetic activation of UAS-*TrpA1*/+; *R84H09*-Gal4/+ and UAS-*TrpA1*/*Tsh*-Gal80; *R84H09*-Gal4/+ result in strong arousal to a similar degree (Fig. 5*F*), indicating that brain-specific activation of *R84H09*-Gal4, most probably in ER3d neurons, promotes wakefulness. While GABA signaling from ER3d neurons may not influence baseline sleep patterns, the wake-promoting effects of *R80C07*-Gal4 activation can most likely be attributed to GABAergic ER3d neurons. Our findings outline a role for ER3d activity in driving wakefulness, but future studies will be required to understand the physiological situations in which ER3d neurons might endogenously activate to suppress sleep. Interestingly, ER3d stimulation selectively increases the probability of awakening, thereby ending sleep episodes prematurely and preventing consolidated sleep. It is possible that, in certain contexts, ER3d neurons may function to reset arousal thresholds or gate state transitions without notably affecting sleep quantity.

**Figure 6.**
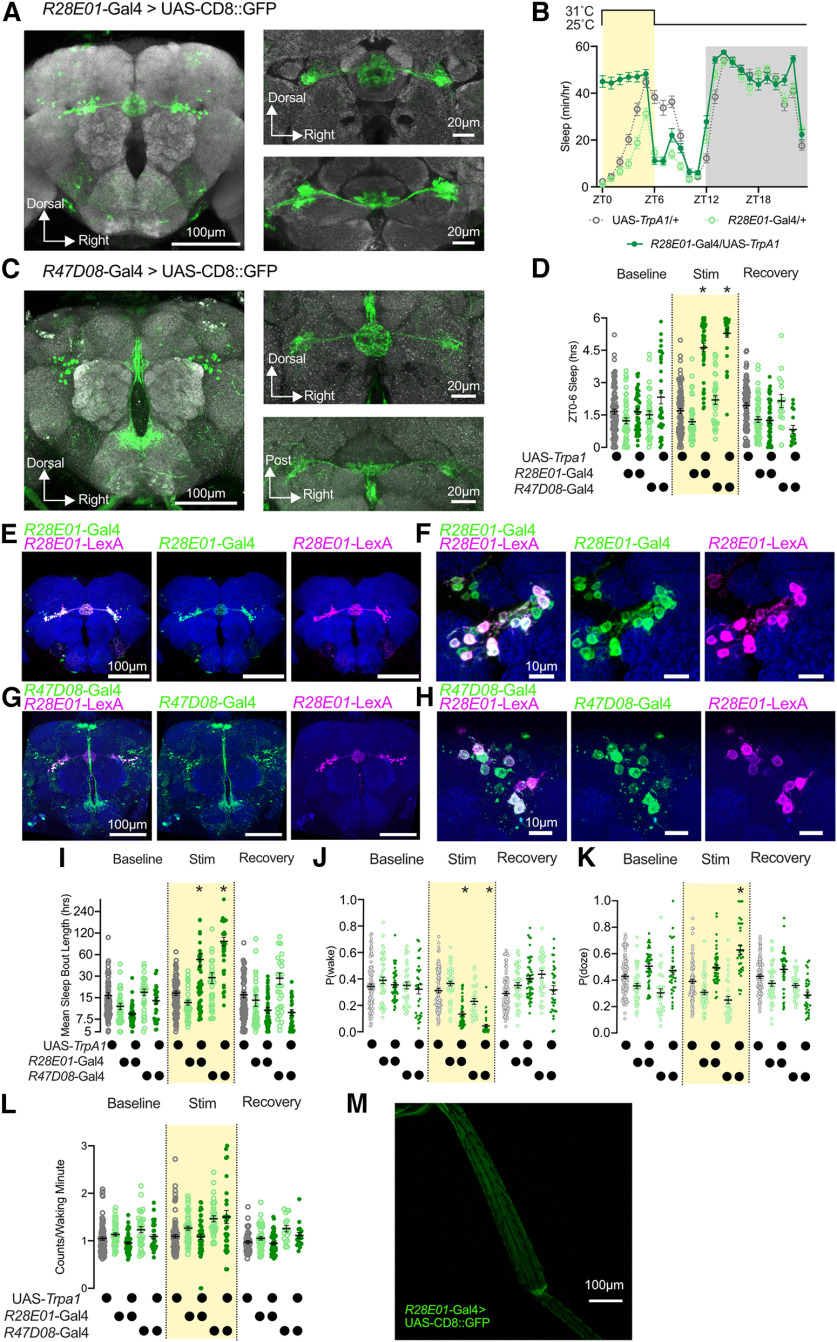
Activation of *R28E01*-Gal4- or *R47D08-*Gal4-expressing neurons acutely increases sleep time and consolidation. ***A***, Expression of CD8::GFP under the control of *R28E01*-Gal4 localizes to the ER3m subpopulation of EB Ring neurons. Left, *z*-Projection of whole-brain confocal scan. Right, Central complex expression of *R28E01*-Gal4 as projections of higher-magnification confocal scans from frontal (top) or dorsal (bottom) views. ***B***, Hourly sleep time course of *R28E01-*Gal4>UAS-*TrpA1* flies (dark green) compared with *R28E01-*Gal4/+ (light green) and UAS-*TrpA1*/+ controls (gray) during the day of a 6 h heat stimulation (31°C) from ZT0 to ZT6. Two-way repeated-measures ANOVA finds a significant time-by-genotype interaction (*F*_(46,3496)_ = 29.46, *p* < 0.0001, *n* = 48–59 flies/group). ***C***, Distribution of CD8::GFP driven by *R47D08*-Gal4. Left, *z*-Projection of whole-brain confocal scan shows labeling in ER3m cells along with the pars intercerebralis and SEZ. Right, Central complex labeling using *R47D08*-Gal4 from frontal (top) and dorsal (bottom) views. ***D***, Sleep time from ZT0 to ZT6 is increased in *R28E01-*Gal4>UAS-*TrpA1* and *R47D08-*Gal4>UAS-*TrpA1* flies (dark green) compared with genetic controls (*R28E01-*Gal4/+ and *R47D08*-Gal4/+ shown in light green; UAS-*TrpA1*/+ in gray) during heat stimulation. No significant difference between experimental (dark green) and genetic controls (light green or gray) could be detected on either baseline or recovery days. Two-way repeated-measures ANOVA finds a significant day-by-genotype interaction (*F*_(8,478)_ = 78.15, *p* < 0.0001, *n* = 30–88 flies/group). ***E***, ***F***, *R28E01*-LexA (magenta) and *R28E01*-Gal4 (green) colabel many of the same ER3m neurons. ***E***, ***F***, Confocal projection through the central brain (***E***) and higher-magnification projection of ER3m cell bodies (***F***). ***G***, ***H***, Both *R47D08*-Gal4 (green) and *R28E01*-LexA (magenta) label overlapping populations of ER3m cells. Confocal projection through central brain (***G***) and ER3m cell bodies (***H*)**. Images in (***G***) were rotated for visual presentation and a black background was extended to fill the rectangular panels. ***I***, The mean length of sleep bouts is elevated in experimental genotypes (*R28E01*-Gal4>UAS-*TrpA1* and *R47D08*-Gal4>UAS-*TrpA1*; dark green) compared with genetic controls (light green) during heat stimulation. Two-way repeated-measures ANOVA finds a significant day-by-genotype interaction (*F*_(8,478)_ = 21.99, *p* < 0.0001, *n* = 30–88 flies/group). ***J***, ***K***, P(wake) (***J***) and P(doze) (***K***) values during ZT0–ZT6 for baseline, heat stimulation, and recovery days for experimental flies (*R28E01*-Gal4/UAS-*TrpA1* and *R47D08*-Gal4/UAS-*TrpA1*; dark green) and genetic controls (UAS-*TrpA1*/+ in gray; *R28E01*-Gal4/+ and *R47D08*-Gal4/+ in light green). Two-way repeated-measures ANOVA finds a significant day-by-genotype effect for P(wake) (*F*_(8,478)_ = 23.90, *p* < 0.0001, *n* = 30–88 flies/group) and P(doze; *F*_(8,478)_ = 23.77, *p* < 0.0001, *n* = 30–88 flies/group). ***L***, Mean counts/waking minute from ZT0 to ZT6 is not changed in *R28E01*-Gal4>UAS-*TrpA1* and *R47D08*-Gal4>UAS-*TrpA1* flies (dark green) during heat stimulation compared with genetic or temperature controls. Two-way repeated-measures ANOVA finds a significant day-by-genotype interaction (*F*_(8,450)_ = 2.895, *p* = 0.0037, *n* = 31–88 flies/group). ***M***, Confocal micrographs of a leg from a *R28E01-*Gal4>UAS-CD8::GFP fly. In all panels, *p < 0.05 by Holm–Šídák's multiple-comparisons test. For whole-brain *z*-stacks used in Figure 6, *A* and *C*, bright autofluorescent debris present in slices above or below the brain were manually erased to prevent blockade of neuronal fluorescence on maximum intensity *z*-projection.

**Figure 7.**
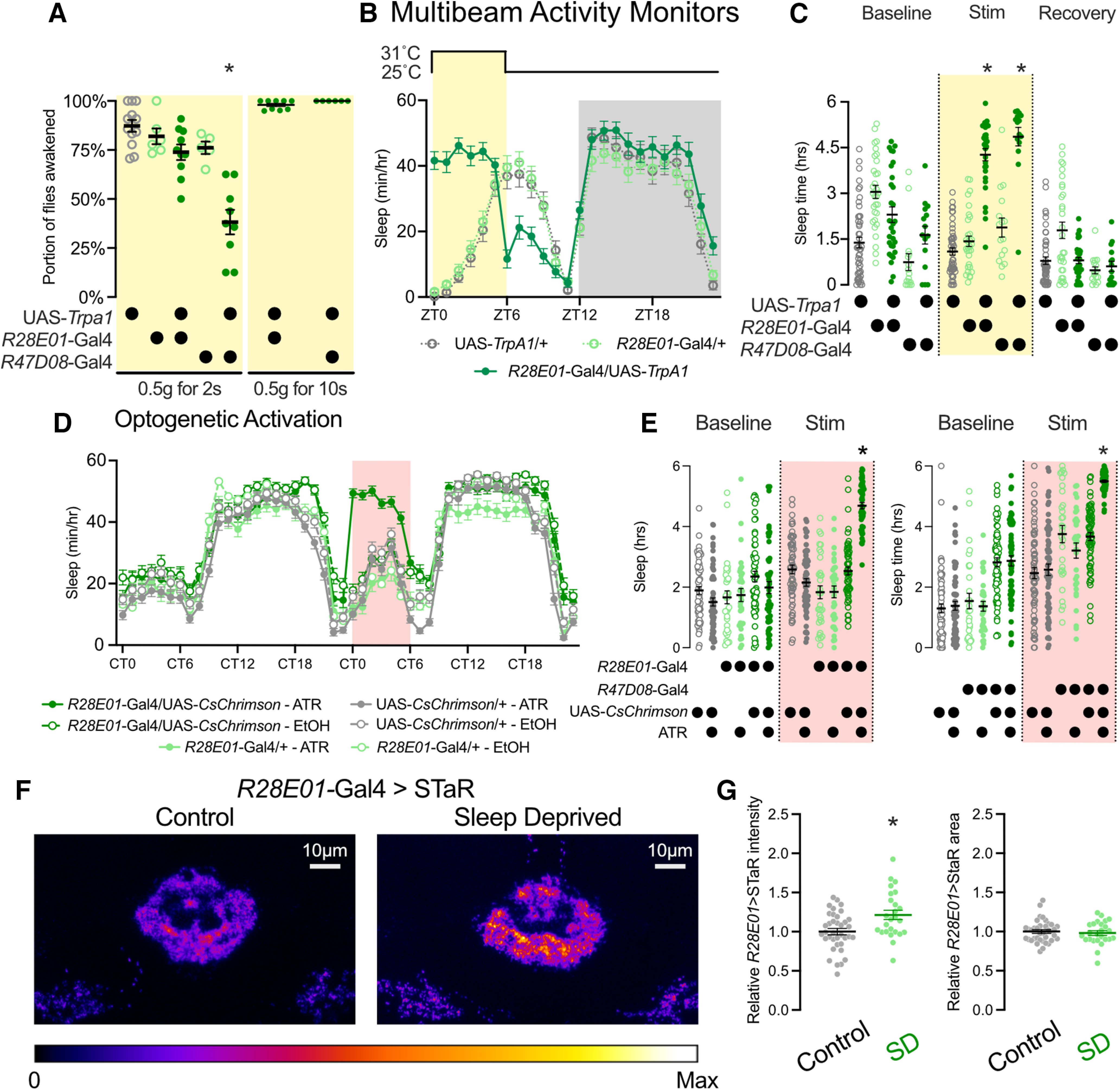
ER3m neurons promote sleep on activation and exhibit presynaptic upscaling after sleep loss. ***A***, Percentage of sleeping flies that were awakened by a brief mechanical vibration (0.5 × *g* for 2 or 10 s). *R28E01*-Ga4/UAS-*TrpA1* and *R47D08*-Gal4/UAS-*TrpA1* experimental flies are depicted in green, genetic controls are shown in gray (UAS-*TrpA1*/+) or light green (*R28E01*-Gal4/+ and *R47D08*-Gal4/+). One-way repeated-measures ANOVA finds a significant effect of genotype for 2 s stimulation (*F*_(4,37)_ = 20.98, *p* < 0.0001, *n* = 5–12 trials/group). Extended mechanical vibration (0.5 × *g* for 10 s) was sufficient to wake both *R28E01*-Ga4/UAS-*TrpA1* and *R47D08*-Gal4/UAS-*TrpA1* flies from sleep when housed at 31°C (*t* test, *p* = 0.064, *t* = 2.024, *n* = 6–9 trials/group). ***B***, Multibeam monitors detect increased sleep in *R28E01-*Gal4>UAS-*TrpA1* flies (dark green) during heat exposure (31°C) from ZT0 to ZT6 compared with *R28E01-*Gal4/+ (light green) and UAS-*TrpA1*/+ (gray) genetic controls. Two-way repeated-measures ANOVA finds a significant time-by-genotype interaction (*F*_(46,2070)_ = 16.58, *p* < 0.0001, *n* = 30–32 flies/group). ***C***, Total sleep time in multibeam monitors from ZT0 to ZT6 is increased in both *R28E01*-Gal4>UAS-*TrpA1* and *R47D08*-Gal4>UAS-*TrpA1* flies (dark green) compared with Gal4/+ (light green) and UAS-*TrpA1*/+ (gray) controls during heat exposure (yellow shading), but not during baseline or recovery days. Two-way repeated-measures ANOVA finds a significant time-by-genotype interaction (*F*_(8,270)_ = 46.03, *p* < 0.0001, *n* = 16–48 flies/group). ***D***, Hourly sleep time course for *R28E01-*Gal4/UAS-*CsChrimson* flies (filled green circles) and genetic/ all-trans retinal (ATR)-free controls during a baseline day and an experimental day with a 6 h red LED illumination from subjective circadian time 0 (CT0; time of lights-on during entrainment) to CT6. Two-way repeated-measures ANOVA finds a significant time-by-group interaction (*F*_(235,13677)_ = 7.58, *p* < 0.0001, *n* = 32–61 flies/group). ***E***, Optogenetic activation of ER3m neurons using *R28E01*-Gal4 (left) or *R47D08-*Gal4 (right) promotes sleep during a 6 h red illumination. Two-way repeated-measures ANOVA finds a significant day-by-group interaction for *R28E01*-Gal4 (*F*_(5,288)_ = 34.09, *p* < 0.0001, *n* = 32–61 flies/group) and for *R47D08*-Gal4 (*F*_(5,312)_ = 16.02, *p* < 0.0001, *n* = 32–64 flies/group). ***F***, Example images from sleep-deprived (right) and control brains (left) showing BRP::smFP_V5 labeling of presynaptic active zones in ER3m neurons using R28E01-Gal4. ***G***, Quantification of relative STaR intensity (left) and cross-sectional area (right) for groups shown in Figure 3*F*. Sleep-deprived brains showed an increase in ER3m>STaR intensity (two-tailed *t* test, *p* = 0.0031; *n* = 25–37 for each group), but no change in area (two-tailed *t* test, *p* = 0.5804, *n* = 25–37 each group). *p < 0.05 by Holm–Šídák’s multiple-comparisons test for ***A***, ***C***, and ***E***; and *p < 0.05 by two-way *t* test for ***G***.

### Sleep-promoting *R28E01*-Gal4 and *R47D08-*Gal4 overlap in ER3m neurons

To precisely characterize the sleep-promoting drivers from our mini-screen, we first confirmed the expression of *R28E01*-Gal4. Confocal projections included in Figure 6*A* indicate that *R28E01*-Gal4 is expressed primarily in ER3m neurons of the ellipsoid body, but also includes a small number of neurons in the suboesophageal zone (Omoto et al., 2018). Heat stimulation only acutely promotes sleep in *R28E01*-Gal4>UAS-*TrpA1* flies compared with genetic controls (*R28E01*-Gal4/+ and UAS-*TrpA1*/+), which rapidly dissipated when flies were returned to 25°C (Fig. 6*B*,*D*). Our mini-screen also identified a second driver, *R47D08*-Gal4, that labels ER3m neurons along with other neuron classes, including cells in the pars intercerebralis (Fig. 6*C*). *R28E01*-Gal4 and *R47D08-*Gal4 both label ER3m populations that also express *R28E01-*LexA (Fig. 6*E–H*), confirming that both Gal4 drivers label overlapping ER3m cells. Over a 6 h activation window from ZT0 to ZT6, activation of both *R28E01-*Gal4>UAS-*TrpA1* and *R47D08*-Gal4>UAS-*TrpA1* significantly increases both sleep time and mean sleep bout duration compared with genetic controls. No difference in either sleep time or bout length was detected between experimental flies and genetic controls on the baseline day before activation or recovery day following activation (Fig. 6*D*,*I*). We next sought to test whether this manipulation might preferentially enhance sleep maintenance with a weaker effect on the initiation of new sleep episodes. To test this possibility, we used a recently described behavioral analysis package to quantify P(wake) or (P(doze) in each minute (Wiggin et al., 2020). As shown in Figure 6, *J* and *K*, stimulation of both *R28E01*-Gal4 and *R47D08*-Gal4 (dark green), resulted in a decreased P(wake) compared both with genetic controls that were also housed at 31°C or with data from the experimental flies that was collected either on the baseline day or recovery day. Experimental *R28E01-*Gal4/UAS-*TrpA1* flies showed little change in P(doze) across the 3 experimental days, while *R47D08*-Gal4*/*UAS-*TrpA1* flies had a significant increase in P(doze) during heat exposure (Fig. 6*J*,*K*). These data indicate that thermogenetic stimulation of *R28E01*-Gal4 and *R47D08*-Gal4 have mixed effects on the initiation of sleep bouts, but strongly suppresses the minute-by-minute probability that a sleeping fly will wake up. Activation of either driver line did not alter activity counts/waking minute (Fig. 6*L*), suggesting that waking locomotor patterns were unchanged during thermogenetic stimulation. Following a recent characterization of wake-promoting sensory neurons in the fly legs (Satterfield et al., 2022), we tested whether *R28E01*-Gal4 labels any peripheral neurons in the leg that might alter arousal or influence locomotion; no GFP-positive somata were detected in the legs of *R28E01-*Gal4>UAS-*CD8::GFP* flies (Fig. 6*M*).

To test whether the increased quiescence observed during *R28E01-*Gal4 or *R47D08-*Gal4 activation fits the same behavioral criteria as spontaneous sleep, we next tested arousability. When flies housed at 31°C were stimulated by a brief vibration (0.5 × *g* for 2 s), we found a reduced percentage of awakenings in *R47D08*-Gal4/UAS-*TrpA1* flies, but no difference between *R28E01-*Gal4/UAS-*TrpA1* flies and genetic controls (Fig. 7*A*, left). An extended 0.5 × *g* stimulus that lasted for 10 s was sufficient to awaken nearly every fly (Fig. 7*A*, right), suggesting that stimulation of both Gal4 drivers elicits a rapidly reversible sleep state. We next sought to validate the sleep-promoting effects of thermogenetic activation of *R28E01-*Gal4 and *R47D08-*Gal4 using alternative behavioral recording and manipulation approaches. We tested the effects of thermogenetic activation using multibeam activity monitors, which provide more precise measurements of movement and positional data than single-beam sensors. Experimental flies exhibited similar increases in quiescence using both systems (Fig. 7*B*,*C*), indicating that the sleep induction that we observed in single-beam monitors was supported by higher-resolution detection of locomotion. Additionally, we also validated the sleep-promoting effects of *R28E01*-Gal4 and *R47D08*-Gal4 using the optogenetic activator *CsChrimson* (Klapoetke et al., 2014). Constant illumination using an array of red LEDs was sufficient to acutely promote sleep in both *R28E01-*Gal4/UAS-*CsChrimson* and *R47D08*-Gal4/UAS-*CsChrimson* flies that were fed all-trans retinal compared with both genetic controls and flies that received vehicle control (Fig. 7*D*,*E*). Next, we sought to further examine whether the sleep-modulatory effects of *R28E01-*Gal4 could be attributed to ER3m neurons. To test whether sleep-promoting ER3m neurons might exhibit structural plasticity in response to sleep loss, we expressed an flp-based reporter for the active zone protein BRP, STaR, using *R28E01*-Gal4 (Kittel et al., 2006; Chen et al., 2014; Peng et al., 2018). As shown in Figure 7, *F* and *G*, ER3m neurons from sleep-deprived flies increased their presynaptic BRP::smFP_V5 by 21.2 ± 6.875% compared with those in rested siblings. ER3m neurons, therefore, may increase presynaptic BRP at times of heightened sleep need. Similar BRP increases have also been identified in ER5 neurons, but not other ER neuron subclasses (Liu et al., 2016; Fig. 3*C*,*D*), indicating that sleep-promoting ER neurons may differ from some other EB neurons in their responses to sleep loss.

### Examining sleep roles of non-ER3m neurons labeled by sleep-promoting drivers

**Figure 8.**
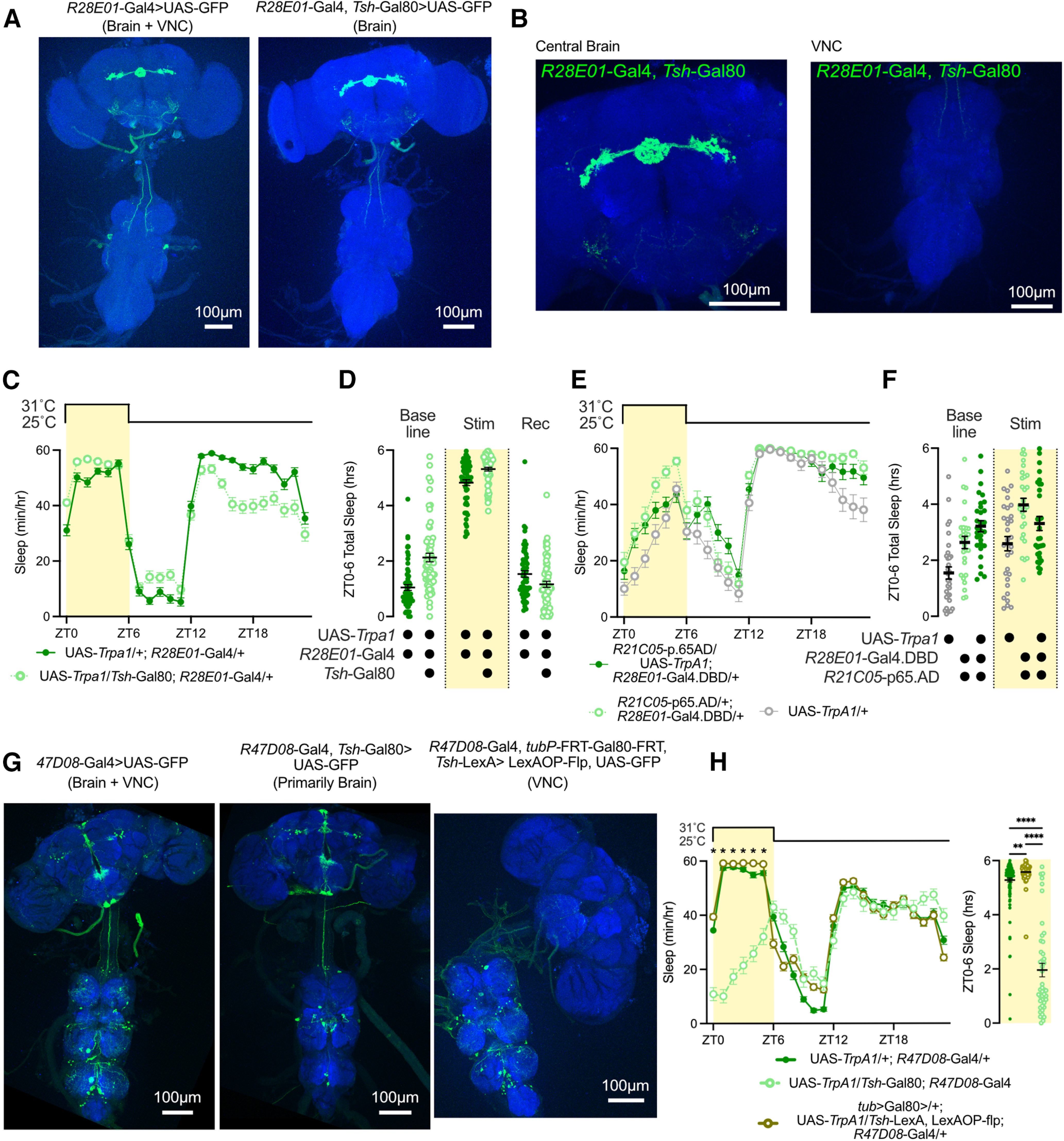
Examining sleep-regulatory neuronal subsets of somnogenic drivers. ***A***, ***B***, CNS expression pattern of UAS-CD8::GFP using *R28E01*-Gal4 alone (left) or with *R28E01*-Gal4 paired with *Tsh-*Gal80 (right). ***B***, Higher-resolution images from the right panel in ***A***. ***C***, Hourly sleep time course for UAS-*TrpA1*/+; *R28E01-*Gal4/+ and UAS-*TrpA1*/*Tsh*-Gal80; *R28E01*-Gal4/+ flies during a day with a 6 h stimulation at 31°C from ZT0 to ZT6. ***D***, Sleep time from ZT0 to ZT6 during a baseline day, stimulation at 31°C, and a recovery day for UAS-*TrpA1*/+; *R28E01-*Gal4/+ and UAS-*TrpA1*/*Tsh*-Gal80 flies. Two-way repeated-measures ANOVA finds a significant time-by-genotype interaction (*F*_(2,374)_ = 18.03, *p* < 0.0001, *n* = 93–96 flies/group). ***E***, ***F***, Sleep time during thermogenetic stimulation from ZT0 to ZT6 in DNg09 descending neurons (dark green) and in genetic controls. Hourly sleep time course shown in ***E*** and ***F*** depicts total sleep from ZT0 to ZT6 during baseline and stimulation days. Two-way repeated-measures ANOVA finds a significant time-by-genotype interaction for both the hourly sleep time course on stimulation day (***E***; *F*_(46,2024)_ = 3.553, *p* < 0.0001, *n* = 29–32), and the ZT0–ZT6 sleep totals for baseline and stimulation day (***F***; *F*_(2,88)_ = 9.78, *p* < 0.0001, *n* = 29–32). ***G***, Brain and VNC expression pattern of UAS-CD8::GFP under the control of *R47D08*-Gal4 (left) or *R47D08*-Gal4 combined with *Tsh*-Gal80 (middle). Right, Brain and VNC confocal stack for *tub*>Gal80>/+; UAS-CD8::GFP/*Tsh*-LexA, LexAOP-flp; *R47D08*-Gal4/+ flies. ***H***, Hourly sleep time courses (left) and total sleep from ZT0 to ZT6 (right) for UAS-*TrpA1*/+; *R47D08*-Gal4/+ (dark green), UAS-*TrpA1*/*Tsh*-Gal80; *R47D08*-Gal4/+ (light green, open circles), and *tub*>Gal80>/+; UAS-*TrpA1*/*Tsh*-LexA, LexAOP-flp; *R47D08*-Gal4/+ (olive green, open circles) flies. All groups were heated to 31°C from ZT0 to ZT6 and returned to 25°C from ZT6 to ZT24. Two-way repeated-measures ANOVA finds a significant time-by-genotype interaction for hourly time course (*F*_(46,5842)_ = 43.09, *p* < 0001, *n* = 44–125 flies/group); significant effect of genotype on total ZT0–ZT6 sleep was found using a nonparametric Kruskal–Wallis test (Kruskal–Wallis statistic = 96.35, *p* < 0.0001, *n* = 44–125 flies/group). Left, *p < 0.05 for pairwise comparisons between UAS-*TrpA1*/*Tsh*-Gal80; *R47D08*-Gal4/+ and both other genotypes using Tukey’s multiple-comparisons test. Right, **p < 0.01, ****p < 0.0001 by Dunn’s pairwise multiple-comparisons test.

In addition to labeling ER3m neurons, we found that *R28E01-*Gal4 is also expressed in a pair of large cell bodies in the VNC (Fig. 8*A*, left). Because peripheral neurons also influence sleep regulation and homeostasis (Seidner et al., 2015; Satterfield et al., 2022), we used a combinatorial strategy to narrow the expression patterns of our identified sleep-promoting and wake-promoting Gal4 drivers. Combining *R28E01-*Gal4 with the Gal4 repressor *Tsh-*Gal80 prevents VNC labeling and weakens SEZ expression, leaving highly specific expression in ER3m neurons (Fig. 8*A*,*B*). Thermogenetic activation of ER3m neurons in UAS-*TrpA1*/*Tsh*-Gal80; *R28E01-*Gal4/+ flies results in a strong increase in sleep that is comparable to the effects of *R28E01*-Gal4 alone (Fig. 8*C*,*D*), indicating a sleep-promoting role for this ER neuron subclass. In addition to ER3m neurons and VNC cells, *R28E01*-Gal4 also labels a small number of neurons in the SEZ. These cells resemble DNg09 descending neurons labeled by a Split-Gal4 driver that includes the *R28E01*-Gal4.DBD hemidriver (SS01058, which includes *R21C05*-p65.AD;*R28E01*-Gal4.DBD; Namiki et al., 2018). Thermogenetic stimulation of *R21C05*-p65.AD;*R28E01*-Gal4.DBD>UAS-*TrpA1* flies did not increase sleep (Figs. 8*E*,*F*), suggesting that these descending neurons may not contribute to the sleep phenotypes that we observe with *R28E01*-Gal4 activation. Together, these findings are consistent with a sleep-promoting role for ER3m neurons.

**Figure 9.**
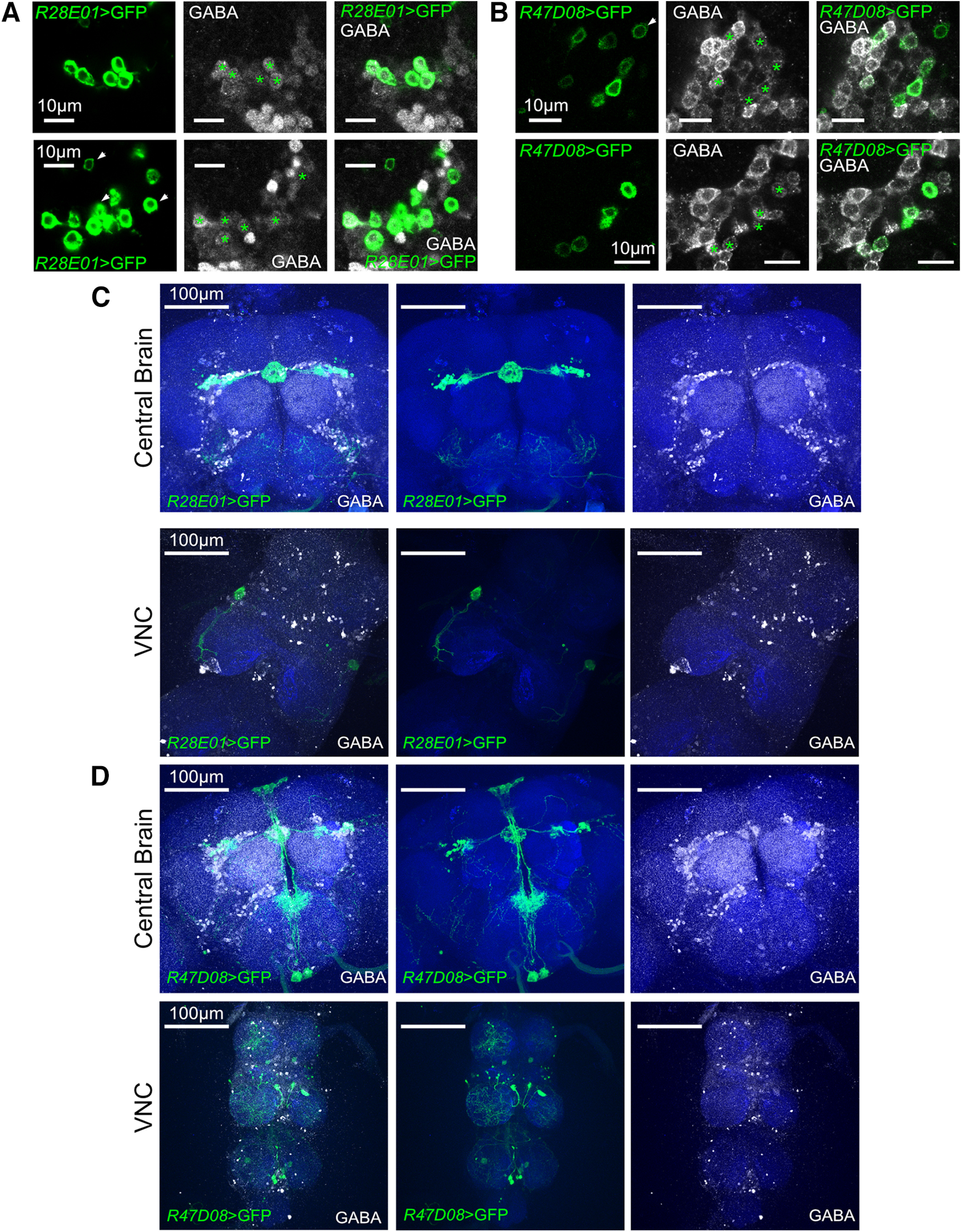
Colocalization of ER3m neurons with GABA immunostaining. ***A***, *R28E01*-positive ER3m soma colocalize with immunostaining for GABA. Top and bottom rows represent distinct confocal slices within an individual *z*-stack. Asterisks indicate GABA-positive ER3m neurons, while arrows represent ER3m cell bodies with no detectable GABA signal. ***B***, UAS-CD8::GFP driven by *R47D08*-Gal4 overlaps with GABA immunostaining. Top and bottom rows represent independent confocal slices; asterisks indicate cell bodies that are positive for both GABA and GFP immunostaining. ***C***, Colocalization of CD8::GFP driven with *R28E01*-Gal4 (green) and GABA immunostaining (white) in the central brain (top row) and VNC (bottom row) is restricted to ER3m neurons. ***D***, Expression of *R47D08*-Gal4>UAS-CD8::GFP (green) in the CNS, with higher-magnification images of the central brain (middle row) and VNC (bottom row). Overlap with GABA immunostaining (white) occurs specifically in ER3m neurons.

As shown in Figures 8*G* and 9*D*, *R47D08*-Gal4 drives expression in ER3m neurons as well as several other neuron types in the central brain and VNC. To test whether the increase in sleep that we observed during *R47D08-*Gal4 activation can be attributed to ER3m neurons or to other cell types, we paired *R47D08*-Gal4 with *Tsh*-Gal80 to reduce expression in the VNC (Fig. 8*G*, left and middle). Combining *Tsh*-Gal80 with *R47D08*-Gal4 reduced the sleep-promoting effect of *TrpA1* activation (Fig. 8*H*), suggesting the presence of sleep-inducing VNC neurons. To directly test for sleep-promoting roles of *R47D08*-positive neurons in the VNC, we used an *flp*-based combination of transgenes to remove central brain expression (Fig. 8*G*, right; Simpson, 2016). Indeed, we found that thermogenetic activation of *R47D08*-positive neurons in the VNC promoted sleep to a similar degree as *R47D08*-Gal4 alone (Fig. 8*H*). Thus, the behavioral outcome of stimulating *R47D08*-Gal4 may reflect the net effect of multiple neuronal populations that include the following: sleep-promoting ER3m neurons; neurosecretory cells in the pars intercerebralis, a major output arm of the circadian clock known to regulate sleep, locomotion, and feeding (Foltenyi et al., 2007; Dus et al., 2015; Barber et al., 2021); and VNC circuits that drive quiescence on stimulation. While our findings suggest that much of the sleep-promoting effect of *R47D08*-Gal4 activation may be generated by VNC neurons, the spatial specificity of *R28E01*-Gal4, especially in combination with *Tsh*-Gal80, suggests that ER3m activation promotes sleep.

### Disrupting GABA synthesis in ER3m neurons dampens injury-induced sleep

**Figure 10.**
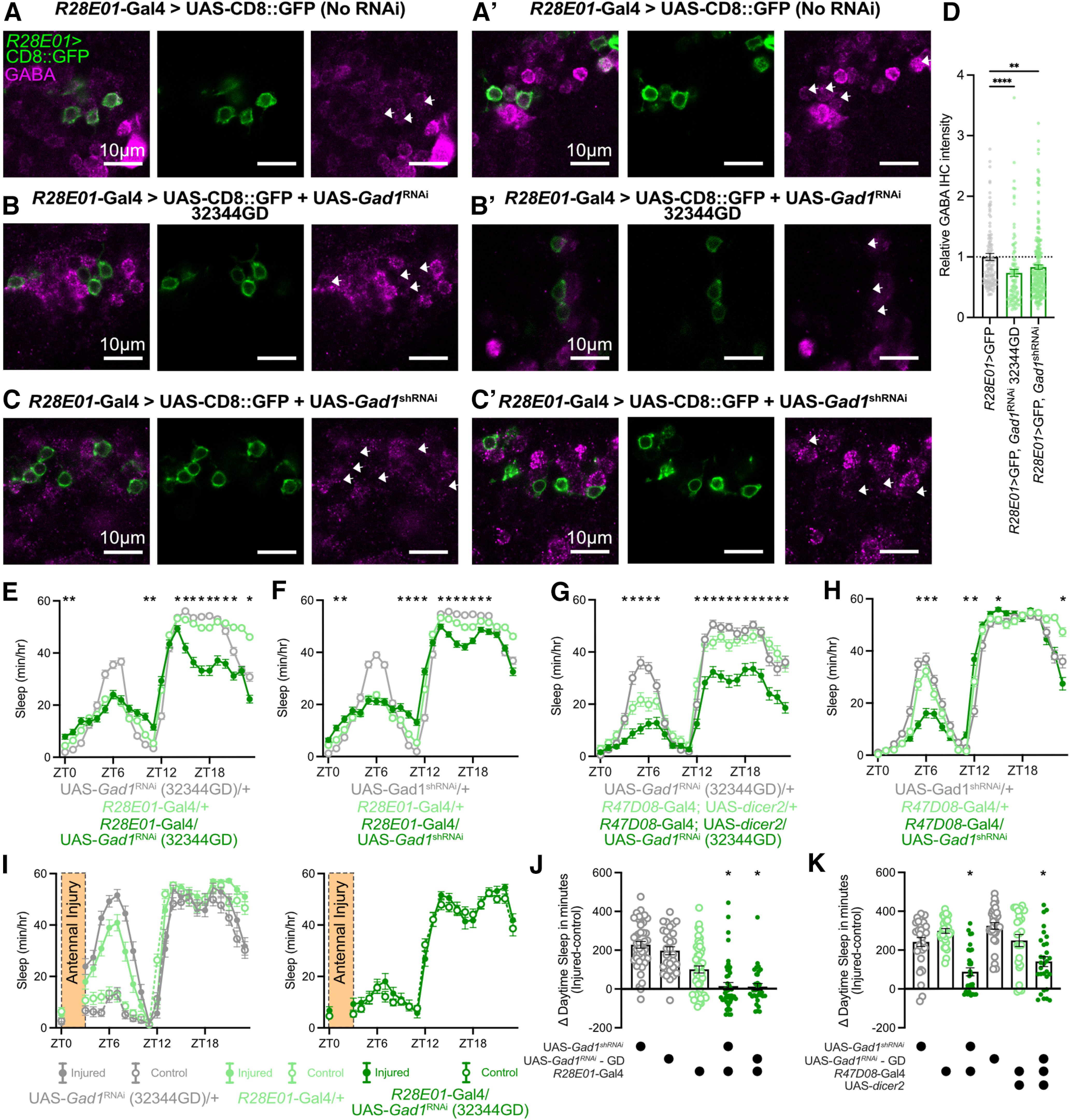
Disruption of GABA synthesis in ER3m neurons impairs sleep regulation. ***A–C′***, Costaining for *R28E01*-Gal4>UAS-CD8::GFP and GABA in flies expressing no *Gad1*^RNAi^ transgene (***A***, ***A′***), UAS-*Gad1*^RNAi^ (32344GD; ***B***, ***B′***), or UAS-*Gad1*^shRNA^ (***C***, ***C′***). For each genotype, two representative confocal slices from separate stacks are shown. Arrows indicate location of GFP-positive cell bodies. ***D***, Relative anti-GABA intensity in GFP-positive ER3m cell bodies of *R28E01*-Gal4>UAS-CD8::GFP controls (gray) or flies using *R28E01-*Gal4 to express UAS-*Gad1*^RNAi^ (32344GD; green, middle) or UAS-*Gad1*^shRNA^ (green, right). Individual points represent single neurons, all data points are normalized to the group mean of *R28E01*-Gal4>UAS-CD8::GFP control flies. Kruskal–Wallis test finds significant effect of genotype (Kruskal–Wallis statistic = 24.24, *p* < 0.0001, *n* = 102–223 cells/group from 5–9 flies/group). **p = 0.0014 by Dunn's multiple-comparisons test, ****p < 0.0001 by Dunn's multiple-comparisons test. ***E–H***, Hourly sleep time courses for genetic controls and flies expressing either of two RNAi constructs targeting *Gad1* in R3m neurons. *R28E01*-driven expression of UAS-*Gad1*^RNAi^ (32344GD) and UAS-*Gad1*^shRNA^ in ***E*** and ***F***, respectively. ***G*** and ***H*** show expression of UAS-*Gad1*^RNAi^ (32344GD) and UAS-*Gad1*^shRNA^ with *R47D08*-Gal4. Two-way repeated-measures ANOVA finds significant time-by-genotype interactions for ***E*** (*F*_(46,9936)_ = 35.72, *p* < 0.0001, *n* = 92–188), ***F*** (*F*_(46,12305)_ = 32.87, *p* < 0.0001, *n* = 159–191), ***G*** (*F*_(46,3887)_ = 8.543, *p* < 0.0001, *n* = 59–64), and ***H*** (*F*_(46,4301)_ = 15.28, *p* < 0.0001, *n* = 63–64). *p < 0.05 by Dunnett’s multiple-comparisons test. ***I***, Sleep during the day of antennal injury for *R28E01*-Gal4/+ and UAS-*Gad1*^RNAi^ (32344GD)/+ flies (left) and for *R28E01*-Gal4/UAS-*Gad1*^RNAi^ (right). Injured flies are depicted with filled dots, controls in empty dots. ***J***, ***K***, Change in daytime sleep following antennal injury for *R28E01*-Gal4 (***J***) and *R47D08*-Gal4 (***K***) driven *Gad1* knockdown (dark green) and genetic controls (gray and light green). Kruskal–Wallis test finds significant effects of genotype for *R28E01*-Gal4 (Kruskal–Wallis statistic = 77.49, *p* < 0.001, *n* = 30–48 flies/group). One-way ANOVAs find significant effects of genotype for *R47D08*-Gal4 (*F*_(5,173)_ = 18.93, *p* < 0.0001, *n* = 24–32 flies/group). *p < 0.05 by Dunn’s multiple-comparisons test (for *R28E01-*Gal4) or Šídák’s multiple-comparisons test (for *R47D08-*Gal4) between experimental genotype and both genetic controls.

To confirm the function of ER3m neurons more precisely, we next addressed their neurochemical output. Previous reports found strong immunolabeling for GABA in the ellipsoid body, suggesting that many ring neurons are likely GABAergic (Hanesch et al., 1989; Zhang et al., 2013; Xie et al., 2017). As depicted in Figure 9, *A* and *B*, our histology confirmed overlap between anti-GABA immunostaining and most, but not all, *R28E01*-positive and *R47D08*-positive cell bodies. Little, if any, overlap occurs outside of ER3m neurons between GABA immunostaining and the expression patterns of *R28E01*-Gal4 or *R47D08-*Gal4 (Fig. 9*C*,*D*). We tested the effect of disrupting GABA production on sleep/wake regulation by expressing two independent RNAi constructs targeting the GABA synthesis enzyme Gad1 in ER3m (Jackson et al., 1990; Dietzl et al., 2007). To validate the efficacy of these RNAi constructs, we used *R28E01*-Gal4 to drive expression of UAS-CD8::GFP along with each RNAi transgene. Flies from each genotype were immunostained for GABA and compared with *R28E01-*Gal4>UAS-CD8::GFP flies that expressed no RNAi. As shown in Figure 10*A–C*, many, but not all, *R28E01-*positive ER3m neurons costained for GABA when no *Gad1* RNAi was expressed (Fig. 10*A*). With the expression of either *Gad1* RNAi transgene, ER3m colocalization with GABA was weakened or eliminated (Fig. 10*B*,*C*). The average anti-GABA signal in *R28E01*-positive ER3m neurons was significantly reduced with either UAS-*Gad1*^RNAi^ 32344GD (mean anti-GABA decreased by 26.31 ± 5.9%) or UAS-*Gad1*^shRNA^ (mean anti-GABA decreased by 16.77 ± 3.7%; Fig. 10*D*). While RNAi knockdown of Gad1 in ER3m neurons using *R28E01*-Gal4 with both effector constructs reduced sleep during the night (Fig. 10*E*,*F*), *R47D08*-driven knockdown yielded mixed results with *R47D08*-Gal4>UAS-*Gad1*^RNAi^ (32344GD) suppressing night sleep (Fig. 10*G*) and *R47D08*-Gal4>UAS-*Gad1*^shRNA^ having little nighttime effect (Fig. 10*H*). Importantly, boosting the efficacy of UAS-*Gad1*^RNAi^ (32344GD) by including UAS-*dicer2* elicited significant wakefulness, indicating that the short-hairpin construct may be less effective. Since these results do not demonstrate uniform sleep disruptions when Gad1 is reduced in ER3m neurons, we next sought to test the necessity of Gad1 within ER3m neurons for promoting sleep at times of high sleep pressure. Our group has previously found that sleep is strongly increased for several hours following antennal transection; this postinjury sleep promotes the removal of presynaptic active zones and plasma membrane from severed olfactory receptor neuron axons (Singh and Donlea, 2020). Given the elevated need for sleep after antennal injury, we tested whether Gad1 expression in ER3m neurons might be required for postinjury sleep responses. Expression of either *Gad1*^RNAi^ construct in *R28E01-*Gal4 (Fig. 10*I*,*J*) or *R47D08-*Gal4 (Fig. 10*K*) was sufficient to weaken the increase in sleep observed during the ∼9 h window after antennal injury. These results indicate that acute activation of ER3m neurons is sufficient to acutely induce sleep, while disrupting GABA production within the same cells prevents flies from increasing their sleep in response to traumatic axotomy.

### Examining sleep effects of stimulating ER3m neurons with Split-Gal4 drivers

**Figure 11.**
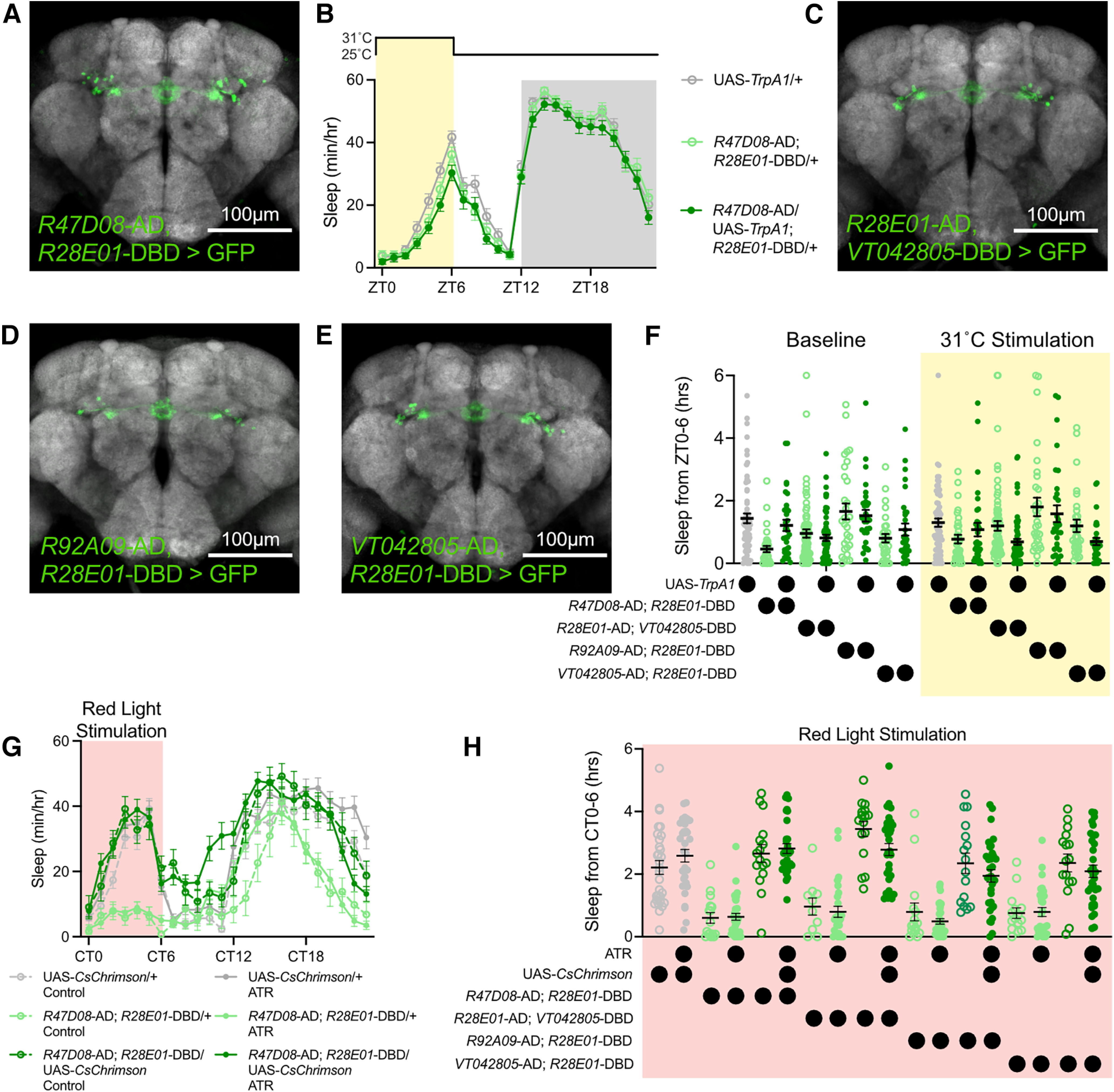
Examining Split-Gal4 drivers labeling ER3m neurons. ***A***, Restricted expression of CD8::GFP in ER3m neurons using *R47D08*-p65.AD; *R28E01*-Gal4.DBD. ***B***, Hourly sleep time course for *R47D08*-p65.AD; *R28E01*-Gal4.DBD > UAS-*TrpA1* (dark green) along with UAS-*TrpA1*/+ (gray) and *R47D08*-p65.AD; *R28E01*-Gal4.DBD/+ (light green) while flies are held at 31°C from ZT0 to ZT6. Two-way repeated-measures ANOVA finds no significant time-by-genotype interaction (*F*_(46,4324)_ = 1.199, *p* = 0.1687, *n* = 63–64 flies/group). ***C–E***, Expression patterns of *R28E01*-p65.AD; *VT042805*-Gal4.DBD (***C***), *R92A09-*p65.AD; *R28E01*-Gal4.DBD (***D***), and *VT042805*-p65.AD; *R28E01*-Gal4.DBD (***E***) in the central fly brain. ***F***, Sleep time from ZT0 to ZT6 during baseline (left) or 31°C stimulation (right) for experimental flies using Split-Gal4 drivers to express UAS-*TrpA1* (dark green) or genetic controls with UAS-TrpA1/+ (gray) or Split-Gal4/+ (light green). Two-way repeated-measures ANOVA detects no significant time-by-genotype interaction (*F*_(8,365)_ = 1.828, *p* = 0.0706, *n* = 30–63 flies/group). ***G***, Sleep time course for *R47D08*-p65.AD; *R28E01*-Gal4.DBD > UAS-*CsChrimson* (dark green) and genetic controls UAS-*CsChrimson*/+ (gray) and *R47D08*-p65.AD; *R28E01*-Gal4.DBD/+ (light green) while flies were stimulated with red light from subjective circadian time 0 (CT0; time of lights-on during entrainment) to CT6 while otherwise housed in constant darkness. Closed circles show flies fed all-trans retinal (ATR); open circles represent vehicle controls. Two-way repeated-measures ANOVA finds a significant group-by-time interaction (*F*_(115,3473)_ = 6.154, *p* < 0.0001, *n* = 16–32 flies/group). ***H***, Total sleep during red light stimulation from CT0 to CT6 for UAS-*CsChrimson*/+ (gray), Split-Gal4/+ (light green), and experimental flies expressing UAS-*CsChrimson* with Split-Gal4s that express in ER3m neurons (dark green). Open circles depict vehicle controls, closed circles show flies fed ATR. One-way ANOVA detects a significant main effect for group (*F*_(17,413)_ = 23.03, *p* < 0.0001, *n* = 16–32 flies/group).

To further examine the effects of ER3m stimulation on sleep, we tested the expression patterns of Split-Gal4 drivers that intersected hemi-drivers under the control of promoters that label ER3m neurons (Jenett et al., 2012; Tirian and Dickson, 2017; Dionne et al., 2018). Combining *R47D08*-p65.AD with *R28E01*-Gal4.DBD restricts expression of UAS-CD8::GFP to the EB (Fig. 11*A*). Thermogenetic activation of this Split-Gal4, however, does not increase sleep (Fig. 11*B*). Similarly, we identified three additional Split-Gal4 combinations using promoter sequences from our initial ER neuron mini-screen that also specifically express in ER3m neurons (Fig. 11*C–E*), but none of these drivers increase sleep on thermogenetic activation (Fig. 11*F*). We further examined the effects of stimulating ER3m-labeling Split-Gal4 drivers using optogenetic activation with *CsChrimson*; these experiments also produced no significant effect of stimulation on sleep amount (Fig. 11*G*,*H*). These results indicate either that the sleep-promoting effects of *R28E01-*Gal4 can be attributed to the following: (1) neurons outside of the ER3m population; (2) the Split-Gal4 drivers tested here not fully reconstituting the collection of *R28E01*-positive ER3m neurons; or (3) expression levels of *TrpA1* and *CsChrimson* produced by ER3m Split Gal4s not being sufficient to elevate sleep.

**Figure 12.**
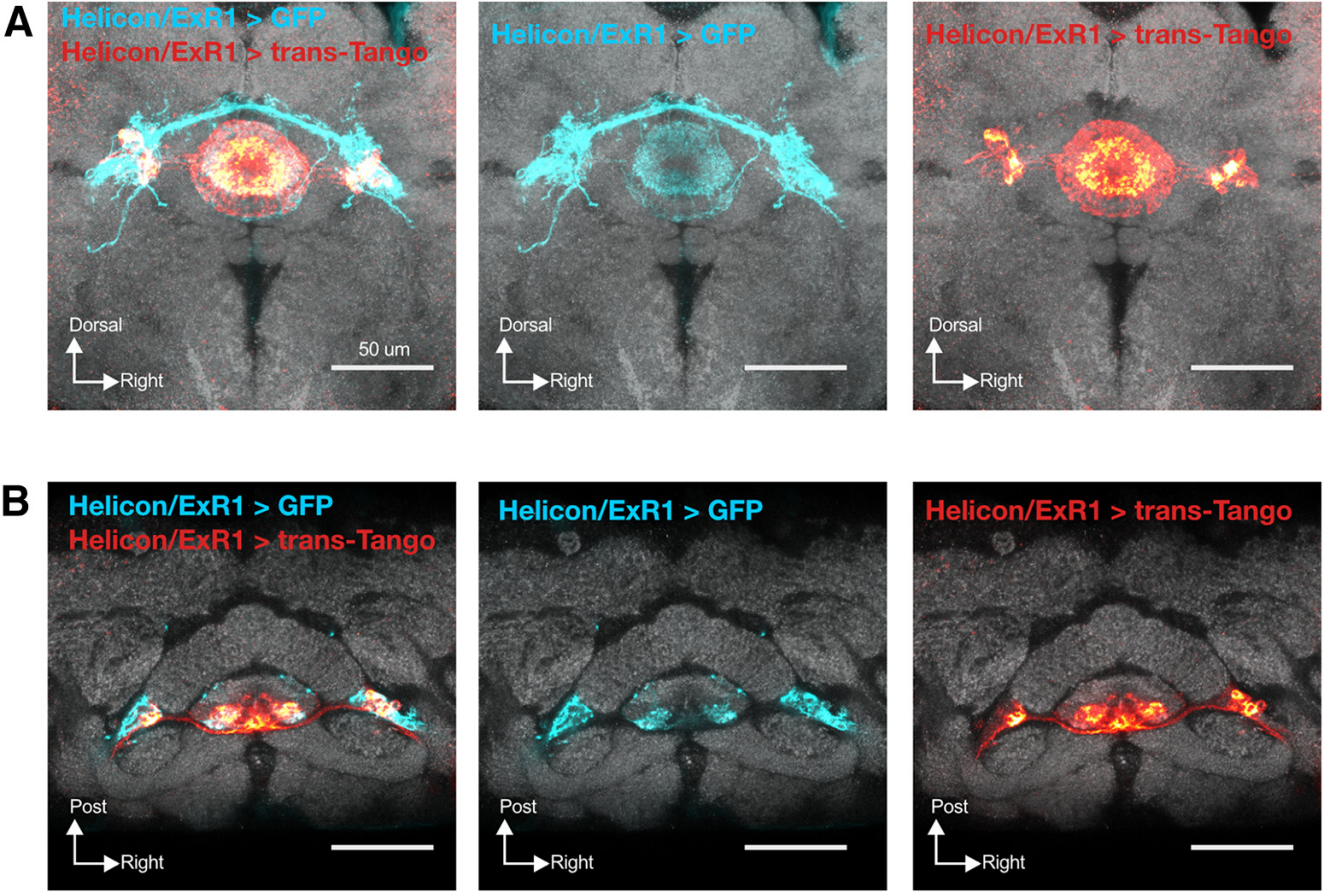
trans-Tango labeling of Helicon/ExR1 neuron postsynaptic targets. ***A***, ***B***, Labeling of Helicon/ExR1 neurons using the intersectional Split-Gal4 driver *R24B11-*p65.AD; *R78A01*-Gal4.DBD (blue) along with trans-Tango tracing of postsynaptic Helicon/ExR1 targets (red). ***A***, ***B***, Frontal view (***A***); dorsal view (***B***).

**Figure 13.**
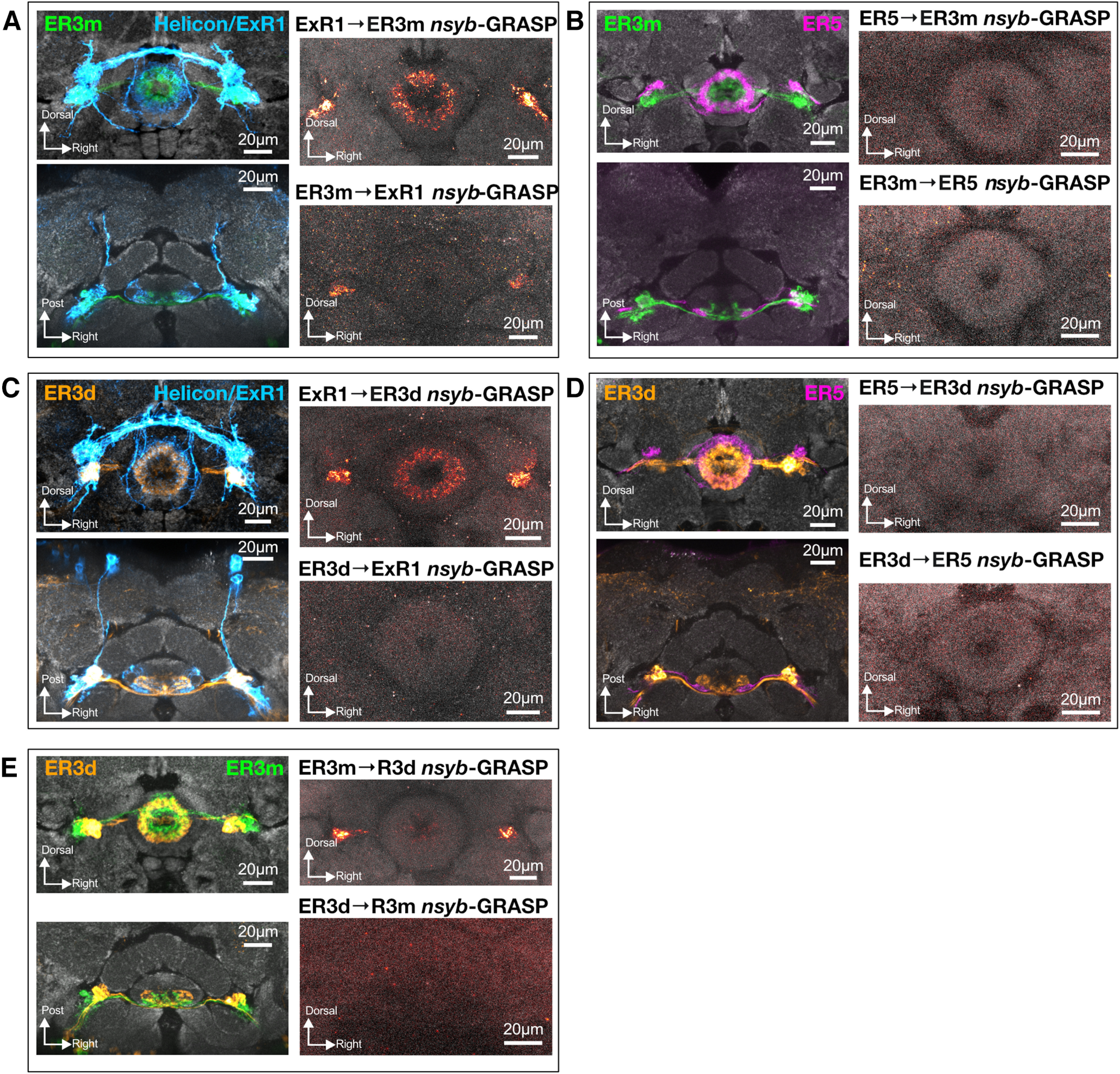
Connectivity between sleep-regulatory neuron types of the ellipsoid body. ***A***, ***B***, ER3m neuron processes (green; *R28E01*-Gal4>UAS-RFP) neighbor neurites from both Helicon/ExR1 (***A***, left panels; blue; *R24B11*-LexA>UAS-GFP) and ER5 (***B***, left panels; magenta; *R48H04*-LexA>UAS-GFP). *nsyb-*GRASP expression reports putative synaptic contacts from Helicon/ExR1 (*R24B11*-LexA) to sleep-promoting ER3m neurons (*R28E01-*Gal4; ***A***, top right), but not from ER5 (*R48H04*-LexA) to ER3m (*R28E01*-Gal4; ***B***, top right). *nsyb*-GRASP fluorescence suggests ER3m (*R80C07*-Gal4) to Helicon/ExR1 (*R24B11*-LexA) contacts in the bulb (***A***, bottom right). Little, if any, signal was found to indicate synaptic connections from ER3m (*R80C07*-Gal4) to ER5 (*R48H04*-LexA; ***B***, bottom right). In ***A*** and ***B***, *R24B11*-LexA was used to label ExR1 cells, *R48H04*-LexA for ER5 neurons, and *R28E01*-Gal4 marked ER3m. ***C***, ***D***, Left panels, Frontal (top) and dorsal (bottom) views of ER3d (orange) neurite localization in the proximity of Helicon/ExR1 (***C***, blue) and ER5 (***D***, magenta) neurons. Neurites from both cell types are in close proximity in the bulb and EB, and *nsyb-*GRASP labeling shows putative synaptic contacts from ExR1 to ER3d neurons (***C***, top right), but we found no GRASP signal from ER5 to ER3d cells (***D***, top right). No evidence was found for connections from ER3d to Helicon/ExR1 (***C***, bottom right) or ER5 (***D***, bottom right) *nsyb-*GRASP. Panels in ***C*** and ***D*** used *R48H04*-LexA to label ER5 neurons, *R24B11*-LexA marked Helicon/ExR1 cells, and ER3d neurons were labeled with *R80C07*-Gal4. ***E***, Left, ER3m (green) and ER3d (orange) innervate closely neighboring zones of the EB and bulb. Using *nsyb*-GRASP, we found evidence for ER3m to ER3d synaptic contacts in the bulb (top right), but no signal representing ER3d–ER3m connections (bottom right). All panels in ***E*** used *R28E01*-LexA to label ER3m neurons and *R80C07*-Gal4 to mark ER3d cells.

### Helicon/ExR1 neurons provide synaptic contacts onto ER3m and ER3d cells

Previous genetic tracing experiments established putative ER neuron output partners (Omoto et al., 2018). However, to more completely characterize EB connectivity in an unbiased fashion, we also expressed a genetically encoded anterograde tracer, trans-Tango (Talay et al., 2017); in Helicon/ExR1 neurons. We found that postsynaptic labeling was restricted solely to ring neurons of the anterior and inner central domains of the EB, which is consistent with direct connections from Helicon/ExR1 to ER5, ER3m, and/or ER3d cells (Fig. 12; Omoto et al., 2018). To test whether ER3m and ER3d neurons might interact with other sleep-regulatory circuitry in the EB, we imaged the proximity of these cells to Helicon/ExR1 and ER5 neurons, which both regulate sleep (Hanesch et al., 1989; Liu et al., 2016; Donlea et al., 2018; Omoto et al., 2018). Neurites from each of these four cell populations (ER3m, ER3d, Helicon/ExR1, and ER5) closely neighbor each other, with all innervating the anterior half of the EB and partitions of the bulb (Fig. 13). We used a genetic reporter for synaptic contacts, GRASP (Feinberg et al., 2008; Macpherson et al., 2015), to test whether each pair of neurons forms putative synaptic contacts. For these studies, we used an activity-dependent GRASP variant that fuses one portion of GFP to the presynaptic vesicle protein nSyb (DiAntonio et al., 1993), while the remaining GFP epitopes were targeted to the plasma membrane of candidate postsynaptic partners, thereby labeling recently active points of contact across the synaptic cleft (Macpherson et al., 2015). Despite the close physical proximity of ER3m, ER3d, ER5, and Helicon/ExR1, robust GRASP signal in the EB could only be found for Helicon/ExR1 → ER3m and Helicon/ExR1 → ER3d connections, while weaker signal was detected for ER3m → ER3d contacts (Fig. 13*A*,*C*,*E*). GRASP labeling for ER3m → ER3d and, more weakly, for ER3m → Helicon/ExR1 contacts was also detected in the bulb (Fig. 13*A*,*E*), consistent with previous trans-Tango tracing for ER3m targets (Omoto et al., 2018). No reliable signal was found to report contacts between ER5 and either ER3m or ER3d (Figs. 13*B*,*D*). These data closely parallel connectivity patterns revealed by detailed reconstructions from serial electron micrographs (Scheffer et al., 2020; Hulse et al., 2021), patch-clamp electrophysiology (Donlea et al., 2018), and genetic-tracing experiments (Omoto et al., 2018). Connectomics mapping at the level of electron microscopy, for instance, found that individual ER5 neurons partner with ER3m or ER3d cells for <0.5% of their identified presynaptic and postsynaptic connections (Scheffer et al., 2020; Hulse et al., 2021).

## Discussion

Our *TrpA1* activation screen identified wake-promoting and sleep-promoting drivers that are expressed in ER neurons, then we more comprehensively examined the contributions of two subclasses: ER3d and ER3m. Activation of two drivers that label ER3d suppresses sleep by impairing sleep maintenance and increasing the probability of awakening without resulting in locomotor hyperactivity. Interestingly, daytime activation of other ER3d-expressing drivers such as VT002226-Gal4 and VT019068-Gal4 results in a modest sleep increase. The factors contributing to these differences are not clear, but recent connectome analysis identified distinct subsets of ER3d neurons with differing synaptic partners (Hulse et al., 2021). These results open the possibility that only a subpopulation of ER3d neurons influences sleep; alternatively, the differing sleep phenotypes could be influenced by neurons other than ER3d cells included in VT002226-Gal4 and VT019068-Gal4. Conversely, two ER3m-expressing Gal4 drivers from our initial Gal4 activation screen, *R28E01*-Gal4 and *R47D08*-Gal4, generated similar increases in sleep on activation. Genetic intersection experiments found that, in addition to ER3m neurons, *R47D08*-Gal4 most likely includes VNC neurons that promote sleep on activation. Complementary loss-of-function experiments that impaired GABA synthesis in ER3m neurons using *R28E01-*Gal4 reduced baseline sleep and prevented flies from responding to traumatic axotomy with an increase in sleep using both *R28E01-*Gal4 and *R47D08*-Gal4. While these results provide some support for a sleep-promoting role for the ER3m neurons labeled by *R28E01*-Gal4, several Split-Gal4 reagents that specifically are expressed in ER3m neurons did not alter sleep time on activation. These results complement another recent study that also found a sleep-promoting effect of ER3m neurons using similar Gal4 drivers (Wei et al., 2023), but future studies of ER3m physiology and influence on sleep may be aided by refined genetic tools to label and address the population of sleep-promoting cells in this subclass.

Although the axons of ER5, ER3m, and ER3d neurons are closely adjacent, our GRASP studies suggest that they are unlikely to be tightly interconnected within the EB. We found no evidence for connections between ER5 and either ER3m or ER3d, or from ER3d to ER3m neurons. While GRASP reported possible contacts from ER3m to ER3d in the bulb, our STaR labeling of presynaptic BRP in ER3m neurons showed little indication of active zones in the bulb. These results are complementary with findings from recent synaptic tracing and connectomics studies (Omoto et al., 2018; Scheffer et al., 2020; Hulse et al., 2021) and suggest that direct synaptic connections between classes of sleep-regulatory ring neurons may be limited. Our data, along with connectome mapping and previous electrophysiology results, suggest that ER5, ER3m, and ER3d each receive synaptic connections from Helicon/ExR1 neurons but, aside from possible ER3m to ER3d contacts, appear to form few contacts across ring neuron types (Donlea et al., 2018; Omoto et al., 2018; Hulse et al., 2021). The mechanisms by which Helicon/ExR1 inputs might modulate activity of ER5, ER3m, and ER3d neurons are unclear, but it is possible that these ER neuron subclasses express distinct complements of postsynaptic receptors that might generate opposing responses to arousal-promoting signals from Helicon/ExR1. It is also possible that GPCRs in distinct ER neuron subclasses could couple with differing classes of G-proteins to produce divergent signaling responses to common Helicon/ExR1 inputs. Intriguingly, both ER5 and ER3m neurons display structural plasticity after prolonged wakefulness (Liu et al., 2016; Ho et al., 2022). While these neurons may be strengthening their outputs to existing synaptic partners, it is alternatively possible that transient synapses may be forming with novel partners in a context-dependent manner. Indeed, ER5 neurons in sleep-deprived flies extend neurites caudally into the EB and increase their connectivity with EPG neurons that encode heading direction (Ho et al., 2022). Future investigations may probe the potential reconfiguration of connectivity between ER neuron subclasses depending on sleep history. Furthermore, it remains unclear whether these ring neuron types communicate via nonsynaptic means, such as volume transmission or gap junctions, act in series via an intermediary, or form parallel sleep regulatory modules.

Analysis of sleep/wake transition probabilities indicates that ER3d stimulation results in fragmented sleep episodes with little effect on the initiation of sleep episodes. Further investigations will be required to identify the precise conditions in which ER3d neurons promote waking. While serotonergic and dopaminergic release onto a collection of ring neuron types can shape sleep architecture and arousal (Lebestky et al., 2009; Liu et al., 2019), our data indicate that acute activation of specific ring neuron populations can drive potent sleep changes. The sleep-regulatory ring neurons that we examine here may be included in the serotonin-responsive and/or dopamine-responsive cell types that are known to influence sleep consolidation or arousability, but the precise physiological effects of neuromodulators on each cell type remain to be examined. The opposing sleep-regulatory effects of ER3d-expressing drivers (Figs. 2-5) and ER5 drivers (Liu et al., 2016; Ho et al., 2022) suggest that parallel representations of sleep-drive and wake-drive may be maintained between different populations of neurons in the EB. These two ring neuron types may represent the primary synaptic targets of arousal-encoding Helicon/ExR1 neurons (Omoto et al., 2018; Hulse et al., 2021), and control of their activity provides new opportunities to probe the organization of sleep control circuitry in the fly. While the downstream targets of ER3d and ER5 neurons are only partially characterized (Hulse et al., 2021; Ho et al., 2022), these cells are positioned in a promising intersection to relay arousal state signals from sleep control neurons to circuits that encode spatial representations of the exterior world (Seelig and Jayaraman, 2013, 2015; Liu et al., 2016; Green et al., 2017; Kim et al., 2017, 2019; Donlea et al., 2018).

## Synthesis

Reviewing Editor: Silvia Pagliardini, University of Alberta Decisions are customarily a result of the Reviewing Editor and the peer reviewers coming together and discussing their recommendations until a consensus is reached. When revisions are invited, a fact-based synthesis statement explaining their decision and outlining what is needed to prepare a revision will be listed below. The following reviewer(s) agreed to reveal their identity: NONE. Note: If this manuscript was transferred from JNeurosci and a decision was made to accept the manuscript without peer review, a brief statement to this effect will instead be what is listed below.

The authors appropriately addressed the final comments and concerns of previous reviewers that evaluated the manuscript in JNeuroscience.

## References

[B1] Barber AF, Fong SY, Kolesnik A, Fetchko M, Sehgal A (2021) Drosophila clock cells use multiple mechanisms to transmit time-of-day signals in the brain. Proc Natl Acad Sci U S A 118:e2019826118. 33658368 10.1073/pnas.2019826118 PMC7958456

[B2] Bausenwein B, Müller NR, Heisenberg M (1994) Behavior-dependent activity labeling in the central complex of Drosophila during controlled visual stimulation. J Comp Neurol 340:255–268. 10.1002/cne.903400210 8201021

[B3] Blum ID, Keleş MF, Baz E-S, Han E, Park K, Luu S, Issa H, Brown M, Ho MCW, Tabuchi M, Liu S, Wu MN (2021) Astroglial calcium signaling encodes sleep need in Drosophila. Curr Biol 31:150–162.e7. 10.1016/j.cub.2020.10.012 33186550 PMC8442851

[B4] Chen Y, Akin O, Nern A, Tsui CYK, Pecot MY, Zipursky SL (2014) Cell-type-specific labeling of synapses in vivo through synaptic tagging with recombination. Neuron 81:280–293. 10.1016/j.neuron.2013.12.021 24462095 PMC4025979

[B6] Deng B, Li Q, Liu X, Cao Y, Li B, Qian Y, Xu R, Mao R, Zhou E, Zhang W, Huang J, Rao Y (2019) Chemoconnectomics: mapping chemical transmission in Drosophila. Neuron 101:876–893.e4. 10.1016/j.neuron.2019.01.045 30799021

[B7] DiAntonio A, Burgess RW, Chin AC, Deitcher DL, Scheller RH, Schwarz TL (1993) Identification and characterization of *Drosophila* genes for synaptic vesicle proteins. J Neurosci 13:4924–4935. 10.1523/JNEUROSCI.13-11-04924.1993 8229205 PMC6576352

[B8] Dietzl G, Chen D, Schnorrer F, Su K-C, Barinova Y, Fellner M, Gasser B, Kinsey K, Oppel S, Scheiblauer S, Couto A, Marra V, Keleman K, Dickson BJ (2007) A genome-wide transgenic RNAi library for conditional gene inactivation in Drosophila. Nature 448:151–156. 10.1038/nature05954 17625558

[B9] Dionne H, Hibbard KL, Cavallaro A, Kao J-C, Rubin GM (2018) Genetic reagents for making Split-GAL4 lines in Drosophila. Genetics 209:31–35. 10.1534/genetics.118.300682 29535151 PMC5937193

[B10] Donelson NC, Donelson N, Kim EZ, Slawson JB, Vecsey CG, Huber R, Griffith LC (2012) High-resolution positional tracking for long-term analysis of Drosophila sleep and locomotion using the “tracker” program. PLoS One 7:e37250. 10.1371/journal.pone.0037250 22615954 PMC3352887

[B11] Dongen HPAV, Maislin G, Mullington JM, Dinges DF (2003) The cumulative cost of additional wakefulness: dose-response effects on neurobehavioral functions and sleep physiology from chronic sleep restriction and total sleep deprivation. Sleep 26:117–126. 10.1093/sleep/26.2.117 12683469

[B12] Donlea JM, Pimentel D, Talbot CB, Kempf A, Omoto JJ, Hartenstein V, Miesenböck G (2018) Recurrent circuitry for balancing sleep need and sleep. Neuron 97:378–389.e4. 10.1016/j.neuron.2017.12.016 29307711 PMC5779612

[B13] Dus M, Lai JS-Y, Gunapala KM, Min S, Tayler TD, Hergarden AC, Geraud E, Joseph CM, Suh GSB (2015) Nutrient sensor in the brain directs the action of the brain-gut axis in Drosophila. Neuron 87:139–151. 10.1016/j.neuron.2015.05.032 26074004 PMC4697866

[B14] Enell L, Hamasaka Y, Kolodziejczyk A, Nässel DR (2007) Gamma-aminobutyric acid (GABA) signaling components in Drosophila: immunocytochemical localization of GABA(B) receptors in relation to the GABA(A) receptor subunit RDL and a vesicular GABA transporter. J Comp Neurol 505:18–31. 10.1002/cne.21472 17729251

[B15] Everson CA (1995) Functional consequences of sustained sleep deprivation in the rat. Behav Brain Res 69:43–54. 10.1016/0166-4328(95)00009-i 7546317

[B16] Everson CA, Bergmann BM, Rechtschaffen A (1989) Sleep deprivation in the rat: III. Total sleep deprivation. Sleep 12:13–21. 10.1093/sleep/12.1.13 2928622

[B17] Feinberg EH, Vanhoven MK, Bendesky A, Wang G, Fetter RD, Shen K, Bargmann CI (2008) GFP Reconstitution Across Synaptic Partners (GRASP) defines cell contacts and synapses in living nervous systems. Neuron 57:353–363. 10.1016/j.neuron.2007.11.030 18255029

[B18] Fisher YE, Lu J, D’Alessandro I, Wilson RI (2019) Sensorimotor experience remaps visual input to a heading-direction network. Nature 576:121–125. 10.1038/s41586-019-1772-4 31748749 PMC7753972

[B19] Foltenyi K, Greenspan RJ, Newport JW (2007) Activation of EGFR and ERK by rhomboid signaling regulates the consolidation and maintenance of sleep in Drosophila. Nat Neurosci 10:1160–1167. 10.1038/nn1957 17694052

[B20] Green J, Adachi A, Shah KK, Hirokawa JD, Magani PS, Maimon G (2017) A neural circuit architecture for angular integration in Drosophila. Nature 546:101–106. 10.1038/nature22343 28538731 PMC6320684

[B21] Guo F, Holla M, Díaz MM, Rosbash M (2018) A circadian output circuit controls sleep-wake arousal in Drosophila. Neuron 100:624–635.e4. 10.1016/j.neuron.2018.09.002 30269992

[B22] Hamada FN, Rosenzweig M, Kang K, Pulver SR, Ghezzi A, Jegla TJ, Garrity PA (2008) An internal thermal sensor controlling temperature preference in Drosophila. Nature 454:217–220. 10.1038/nature07001 18548007 PMC2730888

[B23] Hanesch U, Fischbach KF, Heisenberg M (1989) Neuronal architecture of the central complex in Drosophila melanogaster. Cell Tissue Res 257:343–366. 10.1007/BF00261838

[B24] Ho MCW, Tabuchi M, Xie X, Brown MP, Luu S, Wang S, Kolodkin AL, Liu S, Wu MN (2022) Sleep need-dependent changes in functional connectivity facilitate transmission of homeostatic sleep drive. Curr Biol 32:4957–4966.e5. 10.1016/j.cub.2022.09.048 36240772 PMC9691613

[B26] Hulse BK, Haberkern H, Franconville R, Turner-Evans DB, Takemura S, Wolff T, Noorman M, Dreher M, Dan C, Parekh R, Hermundstad AM, Rubin GM, Jayaraman V (2021) A connectome of the *Drosophila* central complex reveals network motifs suitable for flexible navigation and context-dependent action selection. Elife 10:e66039. 10.7554/eLife.66039 34696823 PMC9477501

[B27] Jackson FR, Newby LM, Kulkarni SJ (1990) Drosophila GABAergic systems: sequence and expression of glutamic acid decarboxylase. J Neurochem 54:1068–1078. 10.1111/j.1471-4159.1990.tb02359.x 1689376

[B28] Jenett A, et al. (2012) A GAL4-driver line resource for Drosophila neurobiology. Cell Rep 2:991–1001. 10.1016/j.celrep.2012.09.011 23063364 PMC3515021

[B29] Kahsai L, Winther ÅME (2011) Chemical neuroanatomy of the Drosophila central complex: distribution of multiple neuropeptides in relation to neurotransmitters. J Comp Neurol 519:290–315. 10.1002/cne.22520 21165976

[B30] Kim SS, Rouault H, Druckmann S, Jayaraman V (2017) Ring attractor dynamics in the Drosophila central brain. Science 356:849–853. 10.1126/science.aal4835 28473639

[B31] Kim SS, Hermundstad AM, Romani S, Abbott LF, Jayaraman V (2019) Generation of stable heading representations in diverse visual scenes. Nature 576:126–131. 10.1038/s41586-019-1767-1 31748750 PMC8115876

[B32] Kittel RJ, Wichmann C, Rasse TM, Fouquet W, Schmidt M, Schmid A, Wagh DA, Pawlu C, Kellner RR, Willig KI, Hell SW, Buchner E, Heckmann M, Sigrist SJ (2006) Bruchpilot promotes active zone assembly, Ca2+ channel clustering, and vesicle release. Science 312:1051–1054. 10.1126/science.1126308 16614170

[B33] Klapoetke NC, et al. (2014) Independent optical excitation of distinct neural populations. Nat Methods 11:338–346. 10.1038/nmeth.2836 24509633 PMC3943671

[B34] Kottler B, Faville R, Bridi JC, Hirth F (2019) Inverse control of turning behavior by dopamine D1 receptor signaling in columnar and ring neurons of the central complex in Drosophila. Curr Biol 29:567–577.e6. 10.1016/j.cub.2019.01.017 30713106 PMC6384123

[B35] Lamaze A, Krätschmer P, Chen K-F, Lowe S, Jepson JEC (2018) A wake-promoting circadian output circuit in Drosophila. Curr Biol 28:3098–3105.e3. 10.1016/j.cub.2018.07.024 30270186

[B36] Lebestky T, Chang J-SC, Dankert H, Zelnik L, Kim Y-C, Han K-A, Wolf FW, Perona P, Anderson DJ (2009) Two different forms of arousal in Drosophila are oppositely regulated by the dopamine D1 receptor ortholog DopR via distinct neural circuits. Neuron 64:522–536. 10.1016/j.neuron.2009.09.031 19945394 PMC2908595

[B37] Liang X, Ho MCW, Zhang Y, Li Y, Wu MN, Holy TE, Taghert PH (2019) Morning and evening circadian pacemakers independently drive premotor centers via a specific dopamine relay. Neuron 102:843–857.e4. 10.1016/j.neuron.2019.03.028 30981533 PMC6533154

[B38] Lin C-Y, Chuang C-C, Hua T-E, Chen C-C, Dickson BJ, Greenspan RJ, Chiang A-S (2013) A comprehensive wiring diagram of the protocerebral bridge for visual information processing in the Drosophila brain. Cell Rep 3:1739–1753. 10.1016/j.celrep.2013.04.022 23707064

[B39] Liu C, Meng Z, Wiggin TD, Yu J, Reed ML, Guo F, Zhang Y, Rosbash M, Griffith LC (2019) A serotonin-modulated circuit controls sleep architecture to regulate cognitive function independent of total sleep in Drosophila. Curr Biol 29:3635–3646.e5. 10.1016/j.cub.2019.08.079 31668619 PMC6832866

[B40] Liu S, Liu Q, Tabuchi M, Wu MN (2016) Sleep drive is encoded by neural plastic changes in a dedicated circuit. Cell 165:1347–1360. 10.1016/j.cell.2016.04.013 27212237 PMC4892967

[B41] Luo J, Shen WL, Montell C (2017) TRPA1 mediates sensation of the rate of temperature change in Drosophila larvae. Nat Neurosci 20:34–41. 10.1038/nn.4416 27749829 PMC5191986

[B42] Macpherson LJ, Zaharieva EE, Kearney PJ, Alpert MH, Lin T-Y, Turan Z, Lee C-H, Gallio M (2015) Dynamic labelling of neural connections in multiple colours by trans-synaptic fluorescence complementation. Nat Commun 6:10024. 10.1038/ncomms10024 26635273 PMC4686661

[B43] Namiki S, Dickinson MH, Wong AM, Korff W, Card GM (2018) The functional organization of descending sensory-motor pathways in Drosophila. Elife 7:e34272. 10.7554/eLife.34272 29943730 PMC6019073

[B44] Ofstad TA, Zuker CS, Reiser MB (2011) Visual place learning in Drosophila melanogaster. Nature 474:204–207. 10.1038/nature10131 21654803 PMC3169673

[B45] Omoto JJ, Keleş MF, Nguyen B-CM, Bolanos C, Lovick JK, Frye MA, Hartenstein V (2017) Visual input to the Drosophila central complex by developmentally and functionally distinct neuronal populations. Curr Biol 27:1098–1110. 10.1016/j.cub.2017.02.063 28366740 PMC5446208

[B46] Omoto JJ, Nguyen B-CM, Kandimalla P, Lovick JK, Donlea JM, Hartenstein V (2018) Neuronal constituents and putative interactions within the Drosophila ellipsoid body neuropil. Front Neural Circuit 12:103. 10.3389/fncir.2018.00103 PMC6278638 30546298

[B47] Peng J, Santiago IJ, Ahn C, Gur B, Tsui CK, Su Z, Xu C, Karakhanyan A, Silies M, Pecot MY (2018) *Drosophila* Fezf coordinates laminar-specific connectivity through cell-intrinsic and cell-extrinsic mechanisms. Elife 7:e33962. 10.7554/eLife.33962 29513217 PMC5854465

[B48] Pfeiffer BD, Ngo T-TB, Hibbard KL, Murphy C, Jenett A, Truman JW, Rubin GM (2010) Refinement of tools for targeted gene expression in Drosophila. Genetics 186:735–755. 10.1534/genetics.110.119917 20697123 PMC2942869

[B49] Pimentel D, Donlea JM, Talbot CB, Song SM, Thurston AJF, Miesenböck G (2016) Operation of a homeostatic sleep switch. Nature 536:333–337. 10.1038/nature19055 27487216 PMC4998959

[B50] Raccuglia D, Huang S, Ender A, Heim M-M, Laber D, Suárez-Grimalt R, Liotta A, Sigrist SJ, Geiger JRP, Owald D (2019) Network-specific synchronization of electrical slow-wave oscillations regulates sleep drive in Drosophila. Curr Biol 29:3611–3621.e3. 10.1016/j.cub.2019.08.070 31630955

[B51] Renn SC, Park JH, Rosbash M, Hall JC, Taghert PH (1999) A pdf neuropeptide gene mutation and ablation of PDF neurons each cause severe abnormalities of behavioral circadian rhythms in Drosophila. Cell 99:791–802. 10.1016/s0092-8674(00)81676-1 10619432

[B52] Satterfield LK, De J, Wu M, Qiu T, Joiner WJ (2022) Inputs to the sleep homeostat originate outside the brain. J Neurosci 42:5695–5704. 10.1523/JNEUROSCI.2113-21.2022 35680412 PMC9302467

[B53] Scheffer LK, et al. (2020) A connectome and analysis of the adult Drosophila central brain. Elife 9:e57443. 10.7554/eLife.57443 32880371 PMC7546738

[B54] Schindelin J, Arganda-Carreras I, Frise E, Kaynig V, Longair M, Pietzsch T, Preibisch S, Rueden C, Saalfeld S, Schmid B, Tinevez J-Y, White DJ, Hartenstein V, Eliceiri K, Tomancak P, Cardona A (2012) Fiji: an open-source platform for biological-image analysis. Nat Methods 9:676–682. 10.1038/nmeth.2019 22743772 PMC3855844

[B55] Seelig JD, Jayaraman V (2013) Feature detection and orientation tuning in the Drosophila central complex. Nature 503:262–266. 10.1038/nature12601 24107996 PMC3830704

[B56] Seelig JD, Jayaraman V (2015) Neural dynamics for landmark orientation and angular path integration. Nature 521:186–191. 10.1038/nature14446 25971509 PMC4704792

[B57] Seidner G, Robinson JE, Wu M, Worden K, Masek P, Roberts SW, Keene AC, Joiner WJ (2015) Identification of neurons with a privileged role in sleep homeostasis in Drosophila melanogaster. Curr Biol 25:2928–2938. 10.1016/j.cub.2015.10.006 26526372 PMC4654679

[B58] Shaw PJ, Tononi G, Greenspan RJ, Robinson DF (2002) Stress response genes protect against lethal effects of sleep deprivation in Drosophila. Nature 417:287–291. 10.1038/417287a 12015603

[B59] Shaw RE, Kottler B, Ludlow ZN, Buhl E, Kim D, Silva SM, da Miedzik A, Coum A, Hodge JJ, Hirth F, Sousa‐Nunes R (2018) In vivo expansion of functionally integrated GABAergic interneurons by targeted increase in neural progenitors. EMBO J 37:e98163. 29728368 10.15252/embj.201798163 PMC6028031

[B60] Simpson JH (2016) Rationally subdividing the fly nervous system with versatile expression reagents. J Neurogenet 30:185–194. 10.1080/01677063.2016.1248761 27846759

[B61] Singh P, Donlea JM (2020) Bidirectional regulation of sleep and synapse pruning after neural injury. Curr Biol 30:1063–1076.e3. 10.1016/j.cub.2019.12.065 32142703 PMC7199647

[B62] Talay M, Richman EB, Snell NJ, Hartmann GG, Fisher JD, Sorkaç A, Santoyo JF, Chou-Freed C, Nair N, Johnson M, Szymanski JR, Barnea G (2017) Transsynaptic mapping of second-order taste neurons in flies by trans-Tango. Neuron 96:783–795.e4. 10.1016/j.neuron.2017.10.011 29107518 PMC5693608

[B63] Tirian L, Dickson BJ (2017) The VT GAL4, LexA, and split-GAL4 driver line collections for targeted expression in the Drosophila nervous system. BioRxiv 198648. 10.1101/198648.

[B64] Vaccaro A, Dor YK, Nambara K, Pollina EA, Lin C, Greenberg ME, Rogulja D (2020) Sleep loss can cause death through accumulation of reactive oxygen species in the gut. Cell 181:1307–1328.e15. 10.1016/j.cell.2020.04.049 32502393

[B66] Wei Y, Hai L, Junwei Y, Wiggin TD, Litao W, Zhiqiang M, Chang L, Griffith LC (2023) Subtype-specific roles of ellipsoid body ring neurons in sleep regulation in *Drosophila*. J Neurosci 43:764–786. 10.1523/JNEUROSCI.1350-22.2022 36535771 PMC9899086

[B67] Wiggin TD, Goodwin PR, Donelson NC, Liu C, Trinh K, Sanyal S, Griffith LC (2020) Covert sleep-related biological processes are revealed by probabilistic analysis in *Drosophila*. Proc Natl Acad Sci U S A 117:10024–10034. 10.1073/pnas.1917573117 32303656 PMC7211995

[B68] Xie X, Tabuchi M, Brown MP, Mitchell SP, Wu MN, Kolodkin AL (2017) The laminar organization of the Drosophila ellipsoid body is semaphorin-dependent and prevents the formation of ectopic synaptic connections. Elife 6:e25328. 10.7554/eLife.25328 28632130 PMC5511011

[B69] Young JM, Armstrong JD (2010) Structure of the adult central complex in Drosophila: organization of distinct neuronal subsets. J Comp Neurol 518:1500–1524. 10.1002/cne.22284 20187142

[B70] Zhang Z, Li X, Guo J, Li Y, Guo A (2013) Two clusters of GABAergic ellipsoid body neurons modulate olfactory labile memory in *Drosophila*. J Neurosci 33:5175–5181. 10.1523/JNEUROSCI.5365-12.2013 23516283 PMC6705003

